# Sesquiterpenes from the Brazilian Red Alga *Laurencia dendroidea* J. Agardh

**DOI:** 10.3390/molecules19033181

**Published:** 2014-03-17

**Authors:** Fernanda Lacerda da Silva Machado, Thatiana Lopes Biá Ventura, Lísia Mônica de Souza Gestinari, Valéria Cassano, Jackson Antônio Lamounier Camargos Resende, Carlos Roland Kaiser, Elena B. Lasunskaia, Michelle Frazão Muzitano, Angélica Ribeiro Soares

**Affiliations:** 1 Instituto de Química, Universidade Federal do Rio de Janeiro, Avenida Athos da Silveira Ramos, 149, 21941-909, Rio de Janeiro, RJ, Brazil; E-Mails: flacerdas@yahoo.com.br (F.L.S.M.); kaiser.ufrj@gmail.com (C.R.K.); 2 Grupo de Produtos Naturais de Organismos Aquáticos (GPNOA), Núcleo de Estudos em Ecologia e Desenvolvimento Sócioambiental de Macaé, Universidade Federal do Rio de Janeiro – Campus Macaé, Av. São José do Barreto, 764, 27965-045, Macaé, RJ, Brazil; E-Mail: lisiagestinari@macae.ufrj.br; 3 Laboratório de Biologia do Reconhecer, Centro de Biociências e Biotecnologia, Universidade Estadual do Norte Fluminense Darcy Ribeiro, Avenida Alberto Lamego, 2000, 28013-602, Campos dos Goytacazes, RJ, Brazil; E-Mails: thativentura@yahoo.com.br (T.L.B.V.); elassounskaia@gmail.com (E.B.L.); 4 Laboratório de Algas Marinhas Professor Édison José de Paula, Departamento de Botânica, Universidade de São Paulo, Rua do Matão, 277, 05508-090, São Paulo, SP, Brazil; E-Mail: vcassano@usp.br; 5 Laboratório Regional de Difração de Raios X (LDRX), Universidade Federal Fluminense, Avenida Litorânea, s/n, 24020-150, Niterói, RJ, Brazil; E-Mail: jresende@id.uff.br; 6 Laboratório de Produtos Naturais, Curso de Farmácia, Universidade Federal do Rio de Janeiro, Campus Macaé, Pólo Novo Cavaleiro – IMMT, Rua Alcides da Conceição, 159, 27933-378, Macaé, RJ, Brazil; E-Mail: mfmuzitano@yahoo.com.br

**Keywords:** chamigrane, *Laurencia dendroidea*, anti-inflammatory, antimycobacterial

## Abstract

Two new chamigrane sesquiterpenes **1**–**2** and three known compounds **3**–**5** were isolated from a lipophilic extract of the red alga *Laurencia dendroidea* collected from the Southeastern Brazilian coast. Dendroidone (**1**) and dendroidiol (**2**) were isolated from samples collected at Biscaia Inlet, Angra dos Reis, Rio de Janeiro and at Manguinhos Beach, Serra, Espírito Santo, respectively. Debromoelatol (**3**), obtusane (**4**) and (1S*,2S*,3S*,5S*,8S*,9S*)-2,3,5,9-tetramethyltricyclo[6.3.0.01.5]undecan-2-ol (**5**) were obtained from specimens collected at Vermelha Beach, Parati, Rio de Janeiro. The structures of new compounds were elucidated by extensive NMR (^1^H-, ^13^C-, COSY, HSQC, HMBC and NOESY) and high resolution mass spectrometry analysis. Additionally, the absolute configuration of compound **2** was assigned by X-ray analysis. Full spectroscopic data is described for the first time for compound **3**. Anti-inflammatory and antimycobacterial activities of compounds **2**–**5** were evaluated. Compounds **3**–**5** inhibited the release of inflammatory mediator NO while TNF-α levels were only affected by **3**. All compounds tested displayed moderate antimycobacterial action.

## 1. Introduction

It is estimated that more than 700 compounds with unique structural features have already been isolated from red algae of genus *Laurencia*, family Rhodomelaceae, order Ceramiales [1,2], which occurs on temperate to tropical shores of the world inhabiting intertidal and subtidal areas [3]. This remarkable chemical diversity comprises sesquiterpenes, diterpenes, triterpenes and acetogenins, mainly halogenated [4,5,6,7,8,9]. Several studies suggest that in the marine environment these compounds have a role as chemical defenses against herbivores, fouling organisms and pathogens [10,11]. Despite the high number of isolated compounds, recent reports confirm the potential of *Laurencia* to produce unknown structures [12]. Chamigrane-type compounds are the main class of sesquiterpenes isolated [2], for which some interesting pharmacological actions are described, including antibacterial [13], cytotoxic [14], and antileishmanial [15]. In addition, *L. undulata* and *L. snackeyi* extracts exhibited anti-inflammatory action [16,17], and the tricyclic brominated diterpene neorogioltriol, isolated from *L. glandulifera*, displayed both *in vivo* and *in vitro* anti-inflammatory activity [18].

The pathogenesis of several diseases such as tuberculosis, caused mainly by *Mycobacterium tuberculosis*, is highly influenced by the inflammatory response. Some anti-inflammatory drugs are employed as an adjunctive therapy for tuberculosis [19]. Therefore, combined anti-inflammatory and antimycobacterial properties in a single compound or class of compounds could be relevant for the treatment of tuberculosis. Furthermore, the long duration of current therapy as well as the associated side effects often compromises its effectiveness and it is intimately linked to the emergence of drug resistance [20]. Thus, there is an urgent need for short and simple regimens, which are both effective and safe.

**Figure 1:**
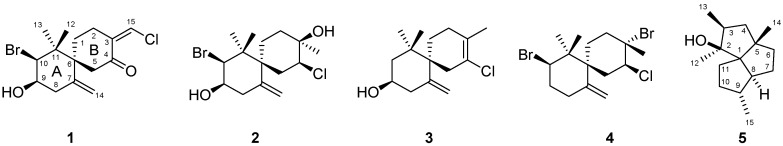
Structures of compounds 1–5.

In our ongoing study on structurally diverse and biologically active compounds from *Laurencia* species, specimens of the Brazilian red alga *L. dendroidea* J. Agardh were collected from three different places along the southeastern coast, and extracted with dichloromethane. Herein we report the extraction, isolation and structure elucidation of compounds **1**–**5** (Figure 1) along with anti-inflammatory and antimycobacterial activities of compounds **2**–**5**.

## 2. Results and Discussion

The organic extracts were subjected to chromatographic separations yielding dendroidone (**1**) from Biscaia Inlet, Angra dos Reis, Rio de Janeiro (population A), dendroidiol (**2**) from Manguinhos Beach, Serra, Espirito Santo (population B) and debromoelatol (**3**), obtusane (**4**) and (1S*,2S*,3S*,5S*,8S*,9S*)-2,3,5,9-tetramethyltricyclo[6.3.0.01.5]undecan-2-ol (5) from Vermelha Beach, Parati, Rio de Janeiro (population C). Compounds **2**–**5** were tested for anti-inflammatory and antimycobacterial activities.

Compound **1** was isolated as an optically active colorless oil, 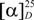 = +6.0° (*c* 0.06, CHCl_3_). The molecular formula was established as C_15_H_20_BrClO_2_ on the basis of HR-APCI-MS data (*m/z* 349.0381 [M+H]^+^, calcd. for C_15_H_21_BrClO_2_, 349.0393), implying five degrees of unsaturation. The infrared (IR) spectrum exhibited absorptions of hydroxyl (3,457 cm^−1^) and carbonyl (1,675 cm^−1^) groups. The ^1^H-NMR spectrum displayed signals corresponding to two methyl groups at δ_H_ 1.09 (H_3_-12) and 1.11 (H_3_-13), two methines geminal to heteroatoms at δ_H_ 4.67 (H-10) and δ_H_ 4.21 (H-9) and three olefinic hydrogens at δ_H_ 5.16 (H-14a), δ_H_ 4.77 (H-14b) and one highly deshielded at δ_H_ 7.24 (H-15), suggesting it was part of a conjugated system. ^13^C and HSQC experiments revealed the presence of two methyls, four aliphatic methylenes, two deshielded methines, two quaternary sp^3^ carbons and five sp^2^ carbon resonances that were assigned to a ketone [δ_C_ 197.6 (C-4)] and two olefins [δ_C_ 117.5 (C-14), 131.3 (C-15), 136.1 (C-3), 142.6 (C-7)]. The positions of the bromine and the hydroxyl groups were deduced from the carbon chemical shifts of carbons at δ_C_ 69.6 (C-10) and 72.0 (C-9), respectively [21]. The presence of an exomethylene group involving C-7 and C-14 was suggested by HMBC correlation between broadened olefinic singlets at δ_H_ 5.16 (H-14a) and δ_H_ 4.77 (H-14b) with carbons at δ_C_ 48.7 (C-6) and 38.6 (C-8). From the ^1^H-^1^H-COSY NMR spectrum the coupling between methylene protons at δ_H_ 2.58 (H_2_-8) and the proton on carbinolic group at δ_H_ 4.21 (H-9) was observed. The latter also couples with the proton on carbon bearing a bromine at δ_H_ 4.67 (H-10). Ring A was established by HMBC correlations from methyls at δ_H_ 1.09 (H_3_-12) and 1.11 (H_3_-13) to the respective carbons [δ_C_ 22.7 (C-13); δ_C_ 20.8 (C-12)] and to δ_C_ 48.7 (C-6), 69.6 (C-10) and 43.0 (C-11).

Ring B was proposed based on correlations between methylenes protons at δ_H_ 2.81 (H-2a) and δ_H_ 1.76 (H-1b) on the COSY spectrum along with HMBC correlations from δ_H_ 2.46 (H-5b) to δ_C_ 197.6 (C-4) and from δ_H_ 1.76 (H-1b) to δ_C_ 48.7 (C-6). Based on the ^13^C-NMR data, a chlorine atom was assigned to C-15 [22]. The *Z* configuration of C-3 double bond was proposed from the observation of correlation between H_2_-2/H-15 on NOESY spectrum. The relative configuration was determined by NOESY correlations and ^1^H-^1^H coupling constants. NOE correlations of H-14b to H-5a demonstrated that exomethylene and methylene CH_2_-5 groups were positioned on the same face. Moreover, halomethine proton H-10 displayed correlations to H-1a and H_3_-13 indicating that H-10 occupied an axial position. Based on the axial orientation of H-10 and the small coupling constant of the carbinolic H-9 (3 Hz) it was suggested that it was equatorial, therefore bromine and hydroxyl were in a *cis* configuration. Hence, the combined data established the structure of compound **1** as (*Z*)-10-bromo-15-chloro-11,11-dimethyl-7-methylidenespiro[5.5]undec-3(15)-ene-4-one which represents a new chemical entity, which was trivially named dendroidone.

**Table 1:** ^1^H (500 MHz) and ^13^C (125 MHz) NMR data for compounds **1**–**3** (CDCl_3_, δ in ppm).

No.	1		2		3	
	δ_C_	δ_H_, mult. (*J* in Hz)	δ_C_	δ_H_, mult. (*J* in Hz)	δ_C_	δ_H_, mult. (*J* in Hz)
**1a** **1b**	25.6	2.16 dm (13.6) 1.76 ddd (14.0, 13.6, 4.8)	22.6	2.00 brd (13.0) 1.60 brd (13.0)	25.8	1.89 m 1.50 m
**2a** **2b**	24.2	2.81 m 2.08 m	33.8	1.70 dt (13.0, 4.0, 3.2) 1.20 m	29.5	1.86 m 1.25 m
**3**	136.1	-	70.3	-	124.6	-
**4**	197.6	-	67.4	4.23 dd (11.0, 5.0)	128.2	-
**5a** **5b**	45.5	2.85 dd (17.5, 3.5) 2.46 d (17.5)	35.1	2.10 m	37.5	2.48 m 2.15 m
**6**	48.7	-	50.5	-	46.6	-
**7**	142.6	-	141.9	-	145.2	-
**8a** **8b**	38.6	2.58 d (3.0)	38.5	2.32 dd (14.0, 2.7) 2.51 dd (14.0, 2.7)	41.7	2.45 m 2.16 m
**9**	72.0	4.21 q (3.0)	72.0	4.07 m	67.9	3.76 ddd (16.0, 10.5, 5.0)
**10**	69.6	4.67 d (3.0)	70.7	4.52 d (3.0)	45.9	1.66 m 1.53 m
**11**	43.0	-	44.2	-	37.5	-
**12**	20.8	1.09 s	20.4	1.03 s	23.8	0.84 s
**13**	22.7	1.11 s	24.1	1.06 s	24.8	0.94 s
**14a** **14b**	117.5	5.16 s 4.77 s	116.7	5.25 s 4.97 s	113.5	5.01 s 4.60 s
**15**	131.3	7.24 m	28.5	1.21 s	19.5	1.70 s

Compound **2** was isolated as colorless crystals, 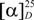 = −19.2° (*c* 0.08, CHCl_3_), with the molecular formula C_15_H_24_BrClO_2_ deduced by HR-ESI-MS from the pseudomolecular ion peak [M+Na]^+^ at *m/z* 375.0417 (calcd. for C_15_H_24_BrClO_2_Na, 375.0525), requiring three degrees of unsaturation. The IR spectrum exhibited absorptions of a hydroxyl group at 3,463 cm^−1^. The ^1^H-NMR spectrum (Table 1) also displayed a pair of broad singlets characteristic of an exocyclic methylene group at δ_H_ 5.25 (H-14a) and 4.97 (H-14b), three tertiary methyl groups at δ_H_ 1.03 (H_3_-13), 1.06 (H_3_-12) and 1.21 (H_3_-15) and three hydrogens of heterosubstituted carbons at δ_H_ 4.07 (H-9), 4.23 (H-4) and 4.52 (H-10). The ^13^C-NMR spectrum and DEPT-135 experiment revealed the presence of fifteen carbon atoms corresponding to three methyls, five methylenes, three methines and four quaternary carbons, including two olefinic carbons and four carbons attached to heteroatoms. ^1^H-^1^H COSY and HMBC correlations indicated that the first ring was similar to compound **1** while the second was proposed based on ^1^H-^1^H COSY correlations between δ_H_ 4.23 (H-4) and 2.10 (2H, m, H_2_-5) and correlations from δ_H_ 1.21 (H_3_-15) to δ_C_ 33.8 (C-2), 70.3 (C-3) and 67.4 (C-4) in HMBC spectrum. Chemical shifts of C-3 and H-4/C-4 indicated the presence of a tertiary alcohol and chlorine substitution, respectively [23].

**Figure 2:**
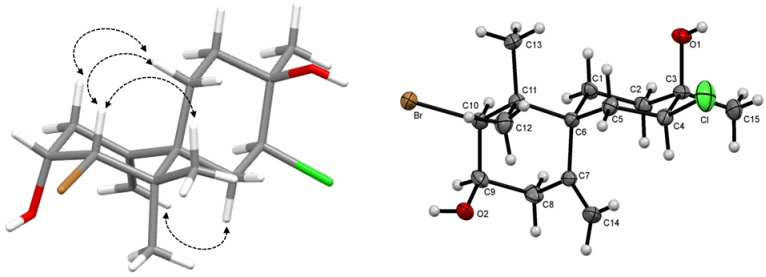
Key NOESY correlations and ORTEP drawing of **2**.

The relative configuration was determined on the basis of measured coupling constants and NOESY spectrum (Figure 2). Taken together, the data suggested that the compound **2** represented a new chamigrane sesquiterpene with chlorohydrin function 4,10-dibromo-4-chloro-3,11,11-trimethyl-7-methylidenespiro[5.5]undec-3,9-diol, for which the trivial name dendroidiol was proposed. Further X-ray crystallographic data of 2 confirmed the suggested structure and defined the absolute configuration as *3R*, *4S*, *6S*, *9R*, *10S* as depicted (Figure 2). The rings A (C1—C2—C3—C4—C5—C6) and B (C7—C8—C9—C10—C11—C12) adopted chair configuration, with ring-puckering parameters q_2_ = 0.060(7)Å; ϕ_2_ = 352(7)° and q_2_ = 0.042(5) Å; ϕ_2_ = 233(7)°, respectively [24].

Compound **3** was isolated as colorless oil. The ^13^C-NMR spectra along with HSQC experiment revealed the presence of fifteen carbons distributed as five quaternary carbons, one methine, six methylenes and three methyls, including four olefinic carbons (Table 1). The molecular formula C_15_H_23_OCl was deduced by NMR and EI-MS data. Like compounds **1** and **2**, the ^1^H-NMR spectrum displayed singlets of an exocyclic methylene group (δ_H_ 5.01, 4.60). Additionally, it also displayed one carbinolic proton at δ_H_ 3.76 and three quaternary methyls (δ_H_ 0.84, 0.94, 1.70). Comparison of NMR spectra of compound **3** to **1** and **2** revealed that ring A differed only on bromine substitution at C-10. The correlations in the HMBC spectrum from methyl H_3_-15 to C-2, C-3 and C-4 indicated the second ring was similar to the described for elatol [15]. Thus, the present data suggested that compound **3** was debromoelatol previously isolated from *L. obtusa* [25]. Full spectroscopic data for compound **3** is described for the first time.

Furthermore, two additional known sesquiterpenes obtusane (**4**) and (1S*,2S*,3S*,5S*,8S*,9S*)-2,3,5,9-tetramethyltricyclo[6.3.0.01.5]undecan-2-ol (**5**) were isolated and identified by comparison of their spectroscopic and physical data to those reported in the literature [15].

Compounds **2**–**5** were submitted to tests evaluating immunomodulatory and antimycobacterial actions (Table 2). These two pharmacological approaches were supported by previous studies reporting immunomodulatory [26] and antimycobacterial activities [27] of *Laurencia* species and also due to the expertise of the group in these two areas.

**Table 2:** IC_50_ values for the compounds isolated from *L. dendroidea* in the production of NO and TNF-α by LPS-stimulated macrophages, against *M. bovis* BCG and in LDH cytotoxicity assay. Values in the same column with different superscript letters (a–d) are significantly different (*p* < 0.05 or *p* < 0.001; results of the Tukey test).

Sample	IC_50_ (μM)			
	NO	TNF-α	*M. bovis* BCG	Cytotoxicity
**2**	>284.3 ^a^	>284.3 ^a^	80.2 ± 4.3 ^a^	>284.3 ^a^
**3**	69.1 ± 4.7 ^b^	133.8 ± 7.4 ^b^	82.4 ± 4.7 ^a^	>392.5 ^b^
**4**	44.9 ± 3.0 ^c^	>250.9 ^c^	44.7 ± 4.0 ^b^	197.2 ± 1.0 ^c^
**5**	74.6 ± 5.8 ^b^	393.4 ± 0.4 ^d^	204.6 ± 7.6 ^c^	416.4 ± 4.9 ^d^
l-NMMA	71.3 ± 4.4 ^b^	-	-	-
Rifampicin	-	-	0.004 ± 1.3 ^d^	-

The *in vitro* anti-inflammatory potential of isolated compounds was evaluated in a preliminary study of immunomodulatory properties, which was assessed by their inhibitory effects on NO and TNF-α productions from LPS-activated RAW 264.7 macrophages. Compounds **3**–**5** inhibited NO release by stimulated macrophages, with IC_50_ values ranging from 44.9 ± 3.0 to 74.6 ± 5.8 μM (Table 2). Compound **4** was significantly more active (*p* < 0.05) while compounds **3** and **5** displayed similar activity to the positive control L-NMMA (L-*N*-monomethyl-arginine), a selective iNOS synthase inhibitor (*p* > 0.05). TNF-α production was moderately inhibited by compound 3 (IC_50_ 133.8 ± 7.4 µM), however, the remaining compounds did not show promising effects.

In the second part of the preliminary pharmacological study, antimycobacterial activity of isolated sesquiterpenes was evaluated using rapidly-growing strain *Mycobacterium bovis* BCG (Table 2). In this test, the sesquiterpene **4** (IC_50_ 44.7 ± 4.0 μM) was the most active compound, but it was less effective than the positive control Rifampicin.

In order to determine whether there was any selectivity, cell viability was assessed by lactate dehydrogenase (LDH) release (Table 2). Compound **4** was considered only moderately toxic while compounds **2**, **3** and **5** showed no toxicity whatsoever.

## 3. Experimental

### 3.1. General Procedures

Optical rotations were measured on a Perkin Elmer model 341LC polarimeter using a Na lamp at 20 °C. IR spectra were obtained with a Perkin Elmer spectrum one FT-IR. ^1^H-NMR, ^13^C-NMR, DEPT-135, COSY, HSQC, HMBC and NOESY spectra were measured employing a Bruker Avance III instrument operating at 500 MHz for ^1^H-NMR and at 125 MHz for ^13^C NMR in CDCl_3_. EI-MS spectra were obtained with a Shimadzu GCMSQP-2010 Plus. HR-APCI-MS spectra were recorded on a MicrOTOF (Bruker Daltonics, Billerica, MA, USA) mass spectrometer. HR-ESI-MS spectra were recorded on an UltrOTOF (Bruker Daltonics) mass spectrometer. Column chromatography was performed with Silicycle SiliaFlash F60 (230–400 mesh) silica and Sephadex LH-20 (Fluka, Steinheim, Germany). Thin layer chromatography was carried out with silica gel GF_254_ plates. The spray reagent was a solution of 2% of Ce(SO_4_)_2_ in H_2_SO_4_. HPLC separations were performed with a Shimadzu instrument equipped with an LC-6AD pump, CBM-20A and SPD-20AV detector using a Shim-pack Prep-ODS, 250 × 20 mm, 5 μm column.

### 3.2. Plant Material

The red seaweed *Laurencia dendroidea* J. Agardh (Rhodomelaceae, Ceramiales) was collected from three distinct areas of the Southeastern Brazilian coast: Biscaia inlet - Angra dos Reis - Rio de Janeiro state (23° 01' S, 44° 14' W), in April, 2011 (Population A); Manguinhos Beach – Serra – Espírito Santo state (20°11' S, 40°11' W), in March, 2010 (Population B) and Vermelha Beach – Parati – Rio de Janeiro state (23°11' S, 44°38' W), in April, 2011 (Population C). Botanical identification was made by L. M. Gestinari and V. Cassano and voucher specimens (Biscaia Inlet: RFA 36068, Manguinhos Beach: RFA 35887 and Vermelha Beach: RFA 36045) were deposited at the Herbarium of the Rio de Janeiro Federal University, Brazil (RFA).

### 3.3. Extraction and Isolation

The air-dried algae of each collection, 476 g (Biscaia Inlet), 422 g (Manguinhos Beach) and 111 g (Vermelha Beach), were extracted three times with CH_2_Cl_2_ (8.0 L, 3.9 L and 2.2 L, respectively) with the assistance of ultrasonication. The solvent was removed under reduced pressure, yielding 12.8 (2.7%), 15.0 (3.5%) and 2.5g (2.3%) of dark green oils, respectively.

Biscaia Inlet crude extract (3.47 g) was separated by silica gel column chromatography (50.5 g) eluted in *n*-hexane–CH_2_Cl_2_ (100:0, 75:25, 50:50; 48:52; 45:55; 40:60; 25:75; 0:100), CH_2_Cl_2_–EtOAc (50:50, 0:100) and MeOH (200 mL of each mixture), resulting in 27 sub-fractions (1–27). Fraction 23 (443 mg), eluted with CH_2_Cl_2_–EtOAc (50:50) was submitted to gel filtration column on Sephadex LH-20 with a mixture of *n*-hexane–CH_2_Cl_2_–MeOH (1:1:1, 250 mL). This process resulted in the separation of six sub-fractions (23.1–23.6). Fraction 23.4 (28 mg) was purified with preparative HPLC (column Shim-pack Prep-ODS, 250 × 20 mm, 5 μm.) using a linear gradient of 50% of CH_3_CN in H_2_O at a flow rate of 20 mL/min and monitoring wavelength of 210 nm, resulting in the isolation of compound **1** (4 mg).

Manguinhos Beach crude extract (4.0 g) was separated by silica gel column chromatography (62.0 g) eluted in *n*-hexane–CH_2_Cl_2_ (100:0, 98:2, 95:5; 90:10, 75:25, 50:50; 0:100), CH_2_Cl_2_–EtOAc (50:50, 0:100) and MeOH (200 mL of each mixture), resulting in 16 sub-fractions (1–16). Fraction 13 (114 mg), eluted with CH_2_Cl_2_, was purified with preparative HPLC (column Shim-pack Prep-ODS, 250 × 20 mm, 5 μm) using a linear gradient of 60% of CH_3_CN in H_2_O at a flow rate of 20 ml/min and monitoring wavelength of 210 nm, resulting in the separation of three sub-fractions (13.1–13.3). Compound **2** (41.0 mg) was identified from fraction 13.1. 

Vermelha Beach crude extract (1.9 g) was purified by silica gel column chromatography (52.0 g) using a mixture of *n*-hexane–CH_2_Cl_2_ (100:0, 95:5, 90:10, 75:25, 50:50, 0:100), CH_2_Cl_2_–EtOAc (50:50, 0:100) and MeOH (200 mL of each mixture). As a result, 24 sub-fractions (1–24) were obtained. Compounds **4** (4.0 mg) and **5** (12.0 mg) were identified in fractions 7 [*n*-hexane–CH_2_Cl_2_ (90:10)] and 13 [*n*-hexane–CH_2_Cl_2_ 50:50], respectively. Fraction 22 (336.0 mg) was submitted to gel filtration column on Sephadex LH-20 with a mixture of *n*-hexane–CH_2_Cl_2_–MeOH (1:1:1, 300 mL). This process resulted on the separation of five sub-fractions (22.1–22.5). Fraction 22.3 (111.0 mg) was further chromatographed in silica gel column chromatography (7.0 g) using a gradient of CH_2_Cl_2_–EtOAc (100:0, 98:2, 95:5, 90:10, 80:20) resulting in five sub-fractions (22.3.1–22.3.5). Compound **3** (23.0 mg) was identified from fraction 22.3.1, eluted in CH_2_Cl_2_.

### 3.4. Spectral Data

*Dendroidone* (**1**). Colorless oil; 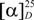 = +6,0° (*c* 0.06, CHCl_3_); IR (film) ν_max_ 3457, 2927, 1675 cm^−1^; ^1^H-NMR and ^13^C-NMR data, see Table 1; EI-MS (rel. int.) *m/z* 320 (2), 318 (2), 313 (3), 311 (2), 308 (3), 306 (15), 304 (12), 269 (3), 267 (9), 249 (26), 221 (19), 207 (75), 183 (33), 175 (22), 171 (24), 157 (23), 147 (38), 143 (59), 133 (27), 131 (28), 129 (35), 119 (66), 117 (32), 115 (34), 107 (50), 105 (63), 93 (32), 91 (96), 85 (95), 83 (41), 79 (58), 77 (61), 41 (100); HR-APCI-MS [M+H]^+^ *m/z* 349.0381 (calcd for C_15_H_20_BrClO_2_, 349.0393).

*Dendroidiol* (**2**). Colorless crystal; m.p. 116 °C; 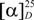 = −19.2° (*c* 0.08, CHCl_3_); IR (film) ν_max_ 3463, 2971, 1918, 1640, 1453, 757, 622 cm^−1^; ^1^H-NMR and ^13^C-NMR data, see Table 1; EI-MS (rel. int.) *m/z* 334 (2), 316 (5), 314 (6), 299 (7), 297 (5), 253 (4), 237 (15), 235 (46), 217 (10), 199 (25), 173 (5), 157 (16), 133 (22), 119 (42), 107(73), 105 (74), 85 (91), 69 (47), 55 (53), 43 (100). HR-ESI-MS [M+Na]^+^ *m/z* 375.0417 (calcd for C_15_H_24_BrClO_2_Na, 375.0525).

*Debromoelatol* (**3**). Colorless oil; 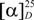 = +9.0° (*c* 0.2, CHCl_3_); IR (film) ν_max_ 3391, 2918, 2849, 1702, 1464, 1296, 939, 758, 720 cm^−1^; ^1^H-NMR and ^13^C-NMR data, see Table 1; EI-MS (rel. int.) *m/z* 254 [M^+^] (1), 239 (15), 237 (6), 236 (34), 223 (10), 221 (29), 219 (6), 210 (8), 209 (4), 208 (25), 201,15 (27), 195 (11), 173 (31), 119 (88), 105 (74), 91 (91), 85 (100).

### 3.5. X-ray Crystallography

X-ray diffraction data was carried out in Nonius Kappa CCD diffractometer at room temperature with radiation MoKα. The collect was performed utilizing Collect software [28] and the data was reduced with EvallCCD [24]. The structure was solved by direct methods and refined by full-matrix least squares on F2 with SHELX-97 package [29]. The positions of hydrogen atoms were generated geometrically and refined according to a riding model. All non-hydrogen atoms were refined anisotropically. The supplementary crystallographic data for **2** reported in this paper have been deposited at the Cambridge Crystallographic Data Center, under the reference number CCDC 950138. Copies of the data can be obtained, free of charge, on application to the Director, CCDC, 12 Union Road, Cambridge CB2 1EZ, UK, fax: +44 1223 336033 or data_request@ccdc.cam.ac.uk.

Compound **2** was crystallized from *n*-hexane to give colorless crystals. Crystal data: C_15_H_24_O_2_ClBr, M = 351.7, colorless block, size 0.30 × 0.26 × 0.16 mm^3^, T = 293(2) K, Orthorhombic, space group P2_1_2_1_2_1_, *a* = 9.2197(8) Å, *b* = 11.2080(8) Å, c = 15.6121(10) Å, V = 1613.3(2) Å^3^, Z = 4, Dc = 1.448 g/cm^3^, µ = 2.71 mm^−1^, F(000) = 728, 14001 reflections measured in the range 3.14° ≤ θ ≤ 25.65°, completeness θ_max_ = 99.7%, 3044 independent, with R_int_ = 0.045; 178 parameters; Final agreement factors: R_1_ = 0.037 [F^2^ > 2σ(F^2^)], wR_2_ = 0.079 and GOOF = 1.09; largest difference peak and hole = 0.73, −0.62 eÅ^−3^. Flack parameter value x = −0.024(13) [30].

### 3.6. Antimycobacterial Activity

Samples were evaluated using a tetrazole salt assay to measure mycobacterial growth in liquid medium [31]. Initially, a suspension of *Mycobacterium bovis* BCG strain Moreau was grown in Middlebrook 7H9 medium supplemented with 0.05% Tween 80 and ADC. At a middle logarithmic growth phase, the bacterial suspension was diluted to obtain a concentration of 2 × 10^7^ CFU/mL, and 50 µL of the resulting suspension was plated in a 96-well plate (1 × 10^6^ CFU/well) and supplemented with 50 µL of each sample in three concentrations. The sealed plate was incubated at 37 °C and 5% CO_2_ for 7 days. After this period, 10 µL of tetrazolium salt (MTT: 3-[4,5-dimethylthiazol-2-yl]-2,5-diphenyltetrazole, Sigma-Aldrich, St. Louis, MO, USA - 5 mg/mL in sterile PBS) was added. After 3 h of incubation, the cells were lysed through the treatment with 100 µL of lyses buffer (20% *w/v* SDS/50% DMF - dimethylformamide in distilled water - pH 4.7). The plate was incubated overnight and measured using a spectrophotometer at 570 nm. As a positive control, a bacterial suspension treated with the standard antimycobacterial drug rifampicin (Sigma-Aldrich-95% purity) at concentrations of 0.0011, 0.0033, 0.01 and 0.03 µg/mL, was used. As a negative control, an untreated bacterial suspension was employed. The test was performed in triplicate and the mean value and standard deviation were calculated.

### 3.7. Determination of Nitric Oxide and TNF-α Production by the RAW 264.7 Macrophage

The murine peritoneal macrophage cell line RAW 264.7 was obtained from the American Type Culture Collection (ATCC, Rockville, MD, USA) and grown at 37 °C and 5% CO_2_ in DMEM F-12 that was supplemented with 10% FCS and gentamicin (50 µg/mL). RAW 264.7 cells (1 × 10^5^ cells/well) were seeded in flat bottom 96-well tissue culture plates (Corning Inc., Corning, NY, USA) in the presence or absence of various concentrations of the samples (100, 20 and 4 µg/mL) and/or LPS (*Escherichia coli* 055:B5; Sigma-Aldrich). After a 24 h incubation period, culture supernatants were collected for NO and TNF-α assays. Nitrite, a stable NO metabolite, was determined by using the Griess test [32]. As a positive control of inhibitory activity, intact, untreated macrophages were used. As a negative control, macrophages stimulated with 1 µg/mL LPS were used. A nitric oxide inhibitor, L-NMMA (Sigma-Aldrich - 98% purity), was also used as a positive control at 20 µg/mL, inhibiting 59.22% ± 2.96% of the NO production. TNF-α was measured by an L929 fibroblast bioassay. This assay system uses murine L929 cells sensitive to TNF-α. For this, murine fibroblast cell line L929 cells (ATCC) (2 × 10^5^ cells/well) were seeded in flat bottom 96-well tissue culture plates (Corning Inc.) 24 h before of being inoculated with macrophage culture supernatant and actinomycin D (2 µg/mL) added. After 24 h of incubation with macrophage culture supernatant, L929 viability was assayed by MTT [3-(4,5-dimethylthiazol-2-yl)-2,5diphenyltetrazolium bromide] method [33]. The cytokine levels were calculated by using a purified recombinant mouse cytokine to obtain a standard curve that correlates cellular viability and TNF-α concentration.

### 3.8. Lactate Dehydrogenase Cytotoxicity Assay

LDH assay was used to evaluate the toxicity of the studied samples towards macrophage cultures. The release of LDH (cytoplasmic enzyme lactate dehydrogenase) from RAW 264.7 cells treated with samples was determined using 50 µL of cell culture supernatant collected 24 h after the treatment, as described in the previous section [34]. The LDH release, which represents an indirect indication of cytotoxicity, was determined colorimetrically using a commercial kit (Doles Reagentes e Equipamentos para Laboratorios Ltda., Goiânia, Brazil). The specific release was calculated as a percentage of the controls: non-treated macrophages as the negative control (O.D. 0.249, cytotoxicity 1.99% ± 0.62%) and 1% Triton X-100 (Vetec Chem., Duque de Caxias, Brazil) detergent treated macrophages as the positive control (O.D. 1.278, cytotoxicity 99.95% ± 2.26%). Final concentrations of DMSO, used as the carrier solvent for the samples, were tested in parallel as a control. Cytotoxicity was shown as percentage of controls. Tests were performed in triplicate and the mean value and standard deviation were calculated.

## 4. Conclusions

In conclusion, the present study resulted in the isolation of five sesquiterpenes from Brazilian specimens of *L. dendroidea*. Compound **1** represents a new compound with a chloroenone group while compound **2** displayed a chlorohydrin function. Full spectroscopic data is described for the first time for compound **3**. Moreover, compound **4** significantly suppressed NO production in LPS-stimulated RAW 264.7 macrophages and also displayed antimycobacterial action against *M. bovis* BCG. Thus, present data showed that *L. dendroidea* is a promising source of immunomodulatory and antimycobacterial drugs.

## Supplementary Materials

Supplementary materials can be accessed at: http://www.mdpi.com/1420-3049/19/3/3181/s1.

## References

[1] Kamada T., Vairappan S. (2012). A new bromoallene-producing chemical type of the red alga *Laurencia nangii* Masuda. Molecules.

[2] Wang B.G., Gloer J.B., Ji N.Y., Zhao J.C. (2013). Halogenated organic molecules of Rhodomelaeae origin: Chemistry and biology. Chem. Rev..

[3] Cassano V., Metti Y., Millar A.J.K., Gil-Rodríguez M.C., Sentíes A., Díaz-Larrea J., Oliveira M.C., Fujii M.T. (2012). Redefining the taxonomic status of *Laurencia dendroidea* (Ceramiales, Rhodophyta) from Brazil and the Canary Island. Eur. J. Phycol..

[4] Blunt J.W., Copp B.R., Hu W.P., Munro M.H.G., Northcote P.T., Prinsep M.R. (2007). Marine natural products. Nat. Prod. Rep..

[5] Blunt J.W., Copp B.R., Keyzers R.A., Munro M.H.G., Prinsep M.R. (2013). Marine natural products. Nat. Prod. Rep..

[6] Cabrita M.T., Vale C., Rauter A.P. (2010). Halogenated compounds from marine algae. Mar. Drugs.

[7] Cen-Pacheco F., Nordström L., Souto M.L., Martín M.N., Fernández J.J., Daranas A.H. (2010). Studies on polyethers produced by red algae. Mar. Drugs.

[8] Dembitsky V.M., Tolstikov G.A. (2004). Natural halogenated sesquiterpenes from marine organisms. Chem. Sustain. Dev..

[9] Díaz-Marrero A.R., Brito I., de la Rosa J.M., D’Croz L., Fabelo O., Ruiz-Perez C., Darias J., Cueto M. (2009). Novel lactone chamigrene-derived metabolites from *Laurencia majuscula*. Eur. J. Org. Chem..

[10] Konig G.M., Wright A.D. (1997). *Laurencia rigida*: Chemical investigations of its antifouling dichloromethane extract. J. Nat. Prod..

[11] Pereira R.C., da Gama B.A., Teixeira V.L., Yoneshigue-Valentin Y. (2003). Ecological roles of natural products of the Brazilian red seaweed *Laurencia obtusa*. Braz. J. Biol..

[12] Li X.D., Ding W., Miao F.P., Ji N.Y. (2012). Halogenated chamigrane sesquiterpenes from *Laurencia okamurae*. Magn. Reson. Chem..

[13] Vairappan C.S., Kawamoto T., Miwa H., Suzuki M. (2004). Potent antibacterial activity of halogenated compounds against antibiotic-resistant bacteria. Planta Med..

[14] Dias T., Brito I., Moujir L., Paiz N., Darias J., Cueto M.J. (2005). Cytotoxic sesquiterpenes from *Aplysia dactylomela*. Nat. Prod..

[15] Machado F.L.S., Pacienza-Lima W., Rossi-Bergmann B., Gestinari L.M.S., Fujii M.T., de Paula J.C., Costa S.S., Lopes N.P., Kaiser C.R., Soares A.R. (2011). Antileishmanial sesquiterpenes from the Brazilian red alga *Laurencia dendroidea*. Planta Med..

[16] Jung W.K., Choi I., Oh S., Park S.G., Seo S.K., Lee S.W., Lee D.S., Heo S.J., Jeon Y.J., Je J.Y. (2009). Anti-asthmatic effect of marine red alga (*Laurencia undulata*) polyphenolic extracts in a murine model of asthma. Food Chem. Toxicol..

[17] Vairappan C.S., Kamada T., Lee W.W., Jeon Y.J. (2013). Anti-inflammatory activity of halogenated secondary metabolites of *Laurencia snackeyi* (Weber-van Bosse) Masuda in LPS-stimulated RAW 264.7 macrophages. J. Appl. Phycol..

[18] Chatter R., Othman R.B., Rabhi S., Kladi M., Tarhouni S., Vagias C., Roussis V., Guizani-Tabbane L., Kharrat R. (2011). *In vivo* and *in vitro* anti-inflammatory activity of neorogioltriol, a new diterpene extracted from the red alga *Laurencia glandulifera*. Mar. Drugs.

[19] McGee S., Hirschmann J. (2008). Use of corticosteroids in treating infectious diseases. Arch. Intern. Med..

[20] Lawn S.D., Zumla A. (2011). Tuberculosis. Lancet.

[21] Wessels M., König G.M., Wright A.D. (2000). New natural product isolation and comparison of the secondary metabolite content of three distinct samples of the sea hare *Aplysia dactylomela* from Tenerife. J. Nat. Prod..

[22] Li X.D., Miao F.P., Liang X.R., Wang B.G., Ji N.Y. (2013). Two halosesquiterpenes from *Laurencia composita*. RSC Adv..

[23] Brennan M.R., Erickson K.L., Minott D.A., Pascoe K.O. (1987). Chamigrane metabolites from a Jamaican variety of *Laurencia obtusa*. Phytochemistry.

[24] Cremer D., Pople J.A. (1975). A general definition of ring puckering coordinates. J. Am. Chem. Soc..

[25] González A.G., Darias J., Diaz A., Fourneron J.D., Martin J.D., Perez C. (1976). Evidence for the biogenesis of halogenated chamigrenes from the red alga *Laurencia obtusa*. Tetrahedron Lett..

[26] Yang E.J., Moon J.Y., Kim M.J., Kim D.S., Kim C.S., Lee W.J., Lee N.H., Hyun C.G. (2010). Inhibitory effect of Jeju endemic seaweeds on the production of pro-inflammatory mediators in mouse macrophage cell line RAW 264.7. J. Zhejiang Univ. Sci. B.

[27] Konig G.M., Wright A.D., Franzblau S.G. (2000). Assessment of antimycobacterial activity of a series of mainly marine derived natural products. Planta Med..

[28] Hooft R.W.W. (1998). Collect Software.

[29] Sheldrick G.M. (2008). A short history of SHELX. Acta Crystallogr..

[30] Flack H.D. (1983). On enantiomorph-polarity estimation. Acta Crystallogr..

[31] Gomez-Flores R., Gupta S., Tamez-Guerra R., Mehta R.T.J. (1995). Determination of MICs for *Mycobacterium avium*-*M. intracellulare* complex in liquid medium by a colorimetric method. Clin. Microbiol..

[32] Da Silva S.A.G., Costa S.S., Rossi-Bergmann B. (1999). The anti-leishmanial effect of *Kalanchoe* is mediated by nitric oxide intermediates. Parasitology.

[33] Mosmann T. (1983). Rapid colorimetric assay for cellular growth and survival: Application to proliferation and citotoxicity assays. J. Immunol. Methods.

[34] Muzitano M.F., Cruz E.A., Almeida A.P., Silva S.A.G., Kaiser C.R., Guette C., Rossi-Bergmann B., Costa S.S. (2006). Quercitrin: na antileishmanial flavonoid glycoside from *Kalanchoe pinnata*. Planta Med..

