# Supplementary material for: Sesquiterpenes from the Brazilian Red Alga Laurencia dendroidea J. Agardh
Source: Molecules. 2014 Mar 17;19(3):3181–92. doi: 10.3390/molecules19033181 (PMC6271906; doi:10.3390/molecules19033181)
Supplement: Supplementary file 1 [file molecules-19-03181-s001.pdf]

Supplementary File

NMR spectra for dendroidone (**1**), dendroidiol (**2**), debrome-elatol (**3**), experimental data for obtusane (**4**) and (1*S**,2*S**,3*S**,5*S**,8*S**,9*S**)-2,3,5,9-tetramethyltricyclo[6.3.0.0$^{1.5}$]undecan-2-ol (**5**). This material is available and free of charge via the Internet.

Figure S1. ^{1}H-NMR (500 MHz, $CDCl_3$) spectrum of compound **1**.

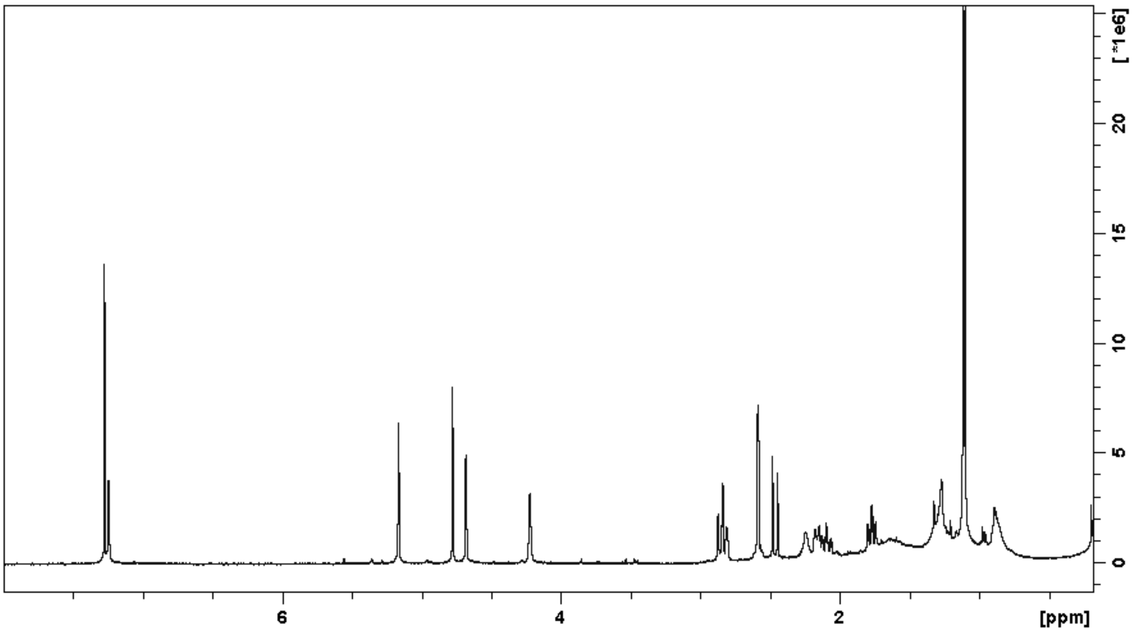

Figure S2. ^{13}C-NMR (125 MHz, $CDCl_3$) spectrum of compound **1**.

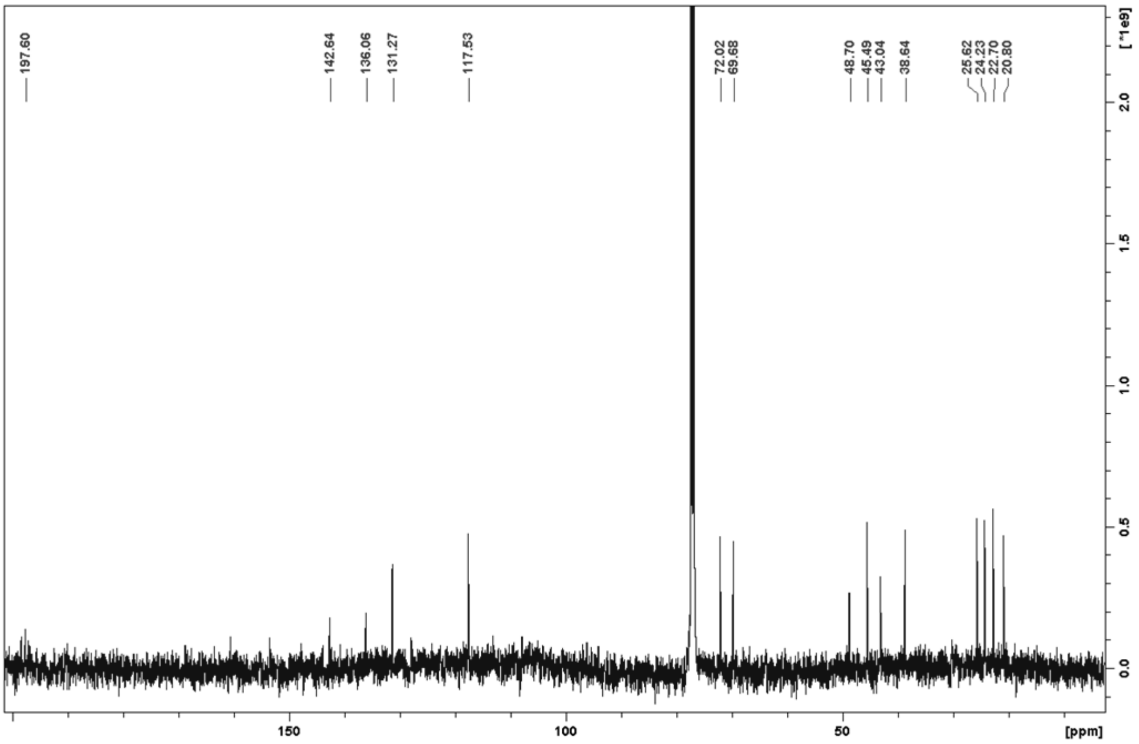

Figure S3. HSQC NMR (125 MHz, $CDCl_3$) spectrum of compound **1**.

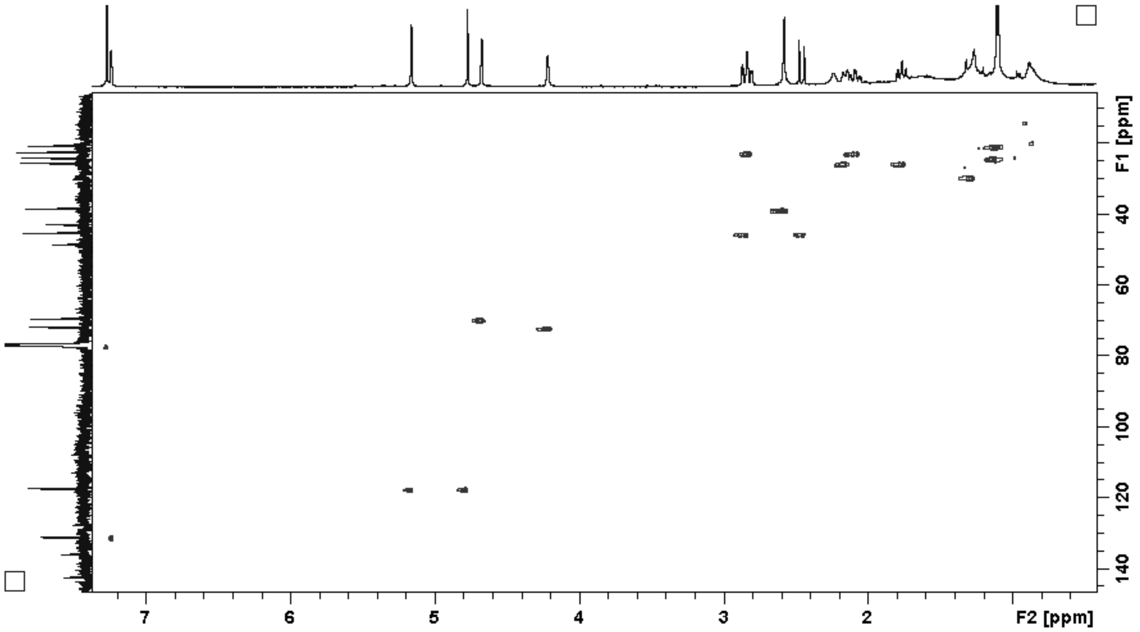

Figure S4. HMBC NMR (125 MHz, $CDCl_3$) spectrum of compound **1**.

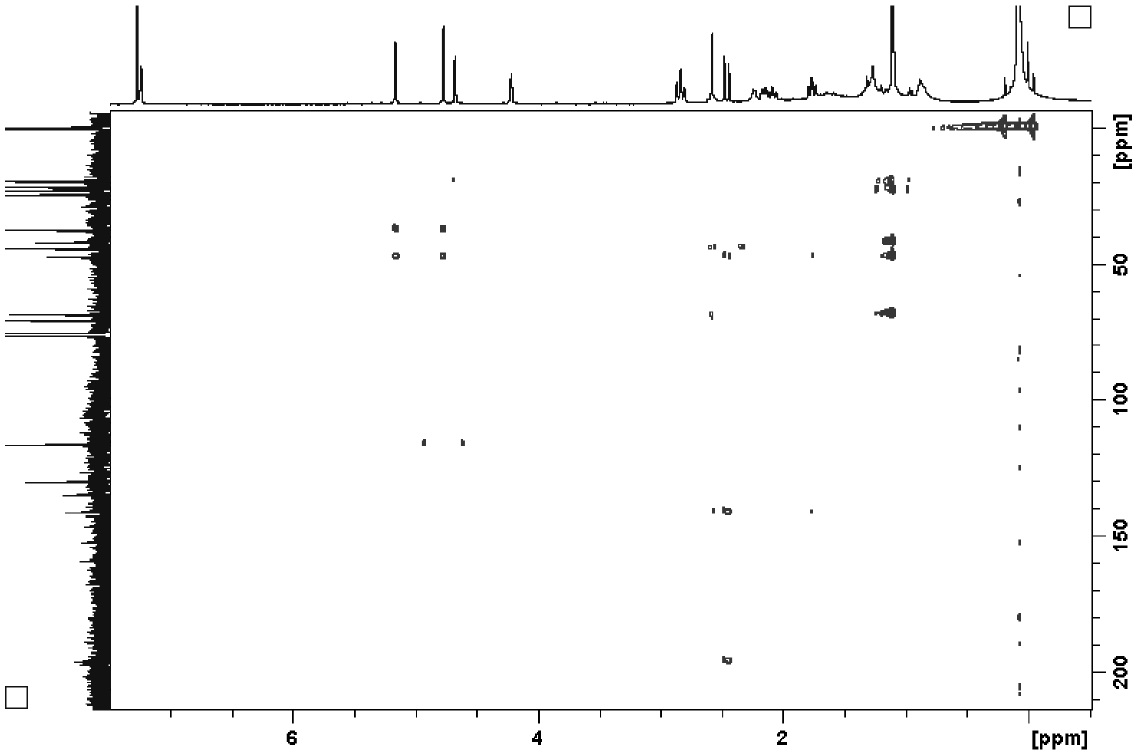

Figure S5. COSY NMR spectrum of compound **1**.

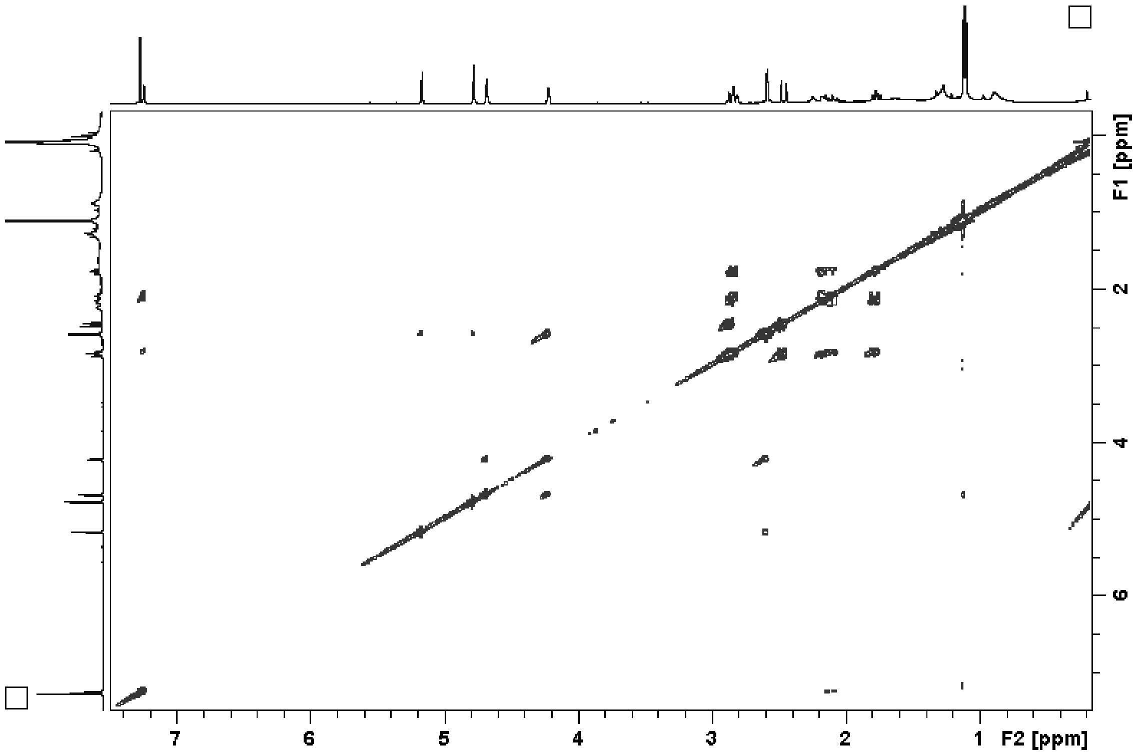

Figure S6. NOESY NMR spectrum of compound **1**.

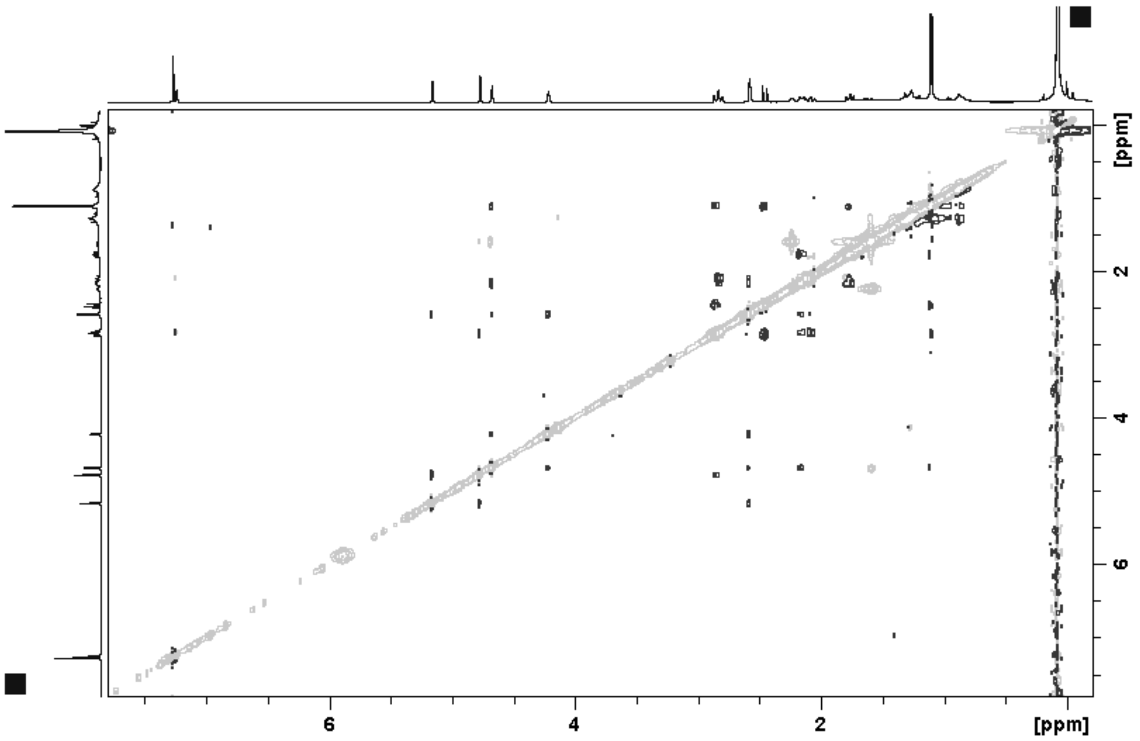

Figure S7. IR spectrum of compound **1**.

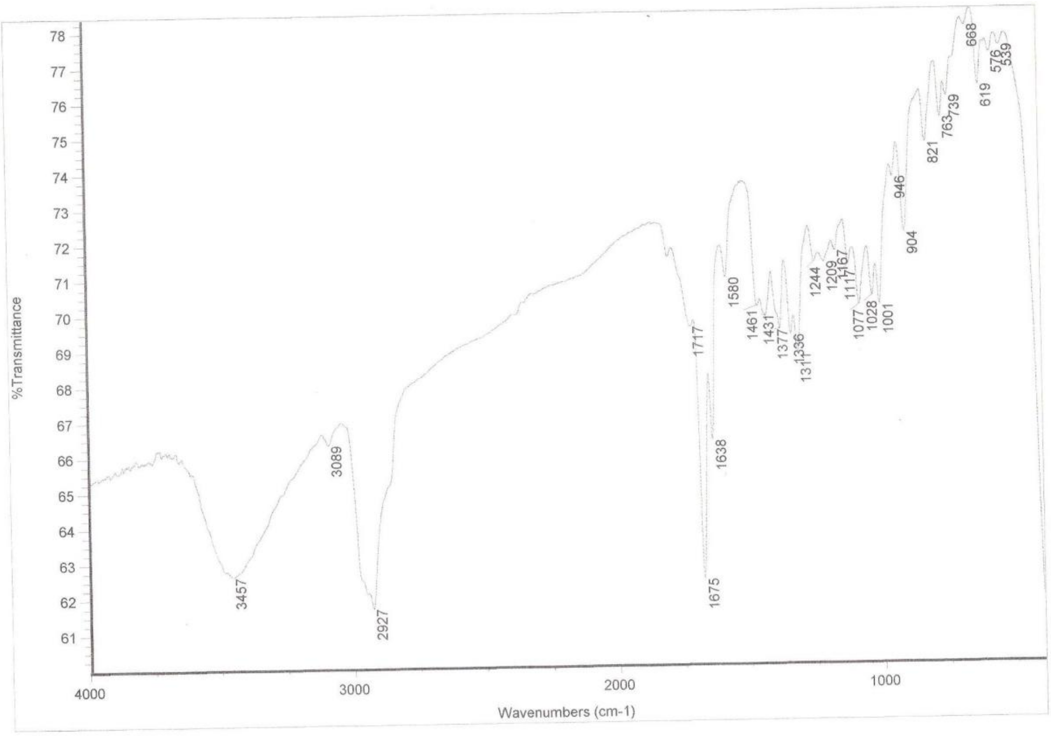

Figure S8. HR-APCI-MS0 (**A**) and EI-MS (**B**) spectra of compound **1**.

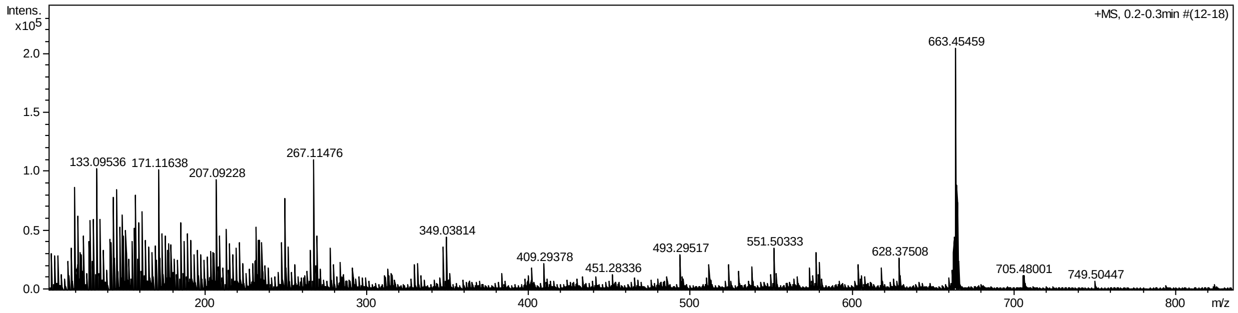

(**A**)

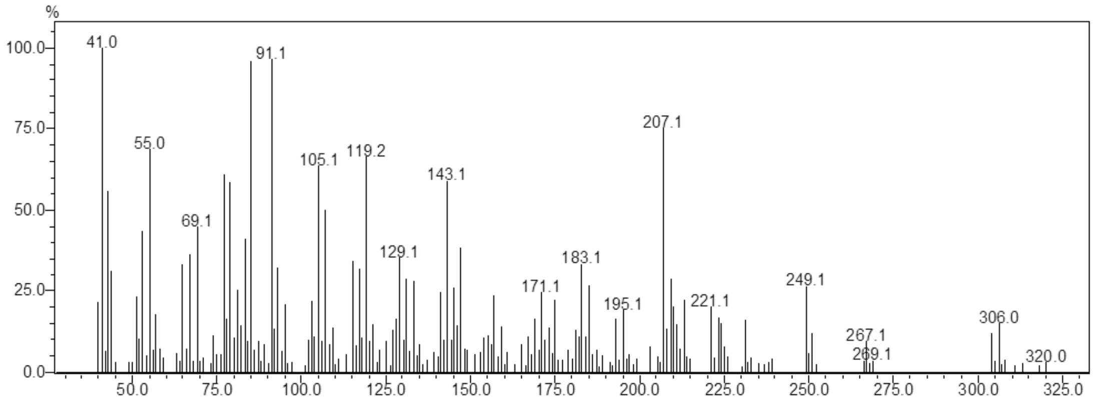

(**B**)

Figure S9. ^{1}H-NMR (500 MHz, $CDCl_3$) spectrum of compound **2**.

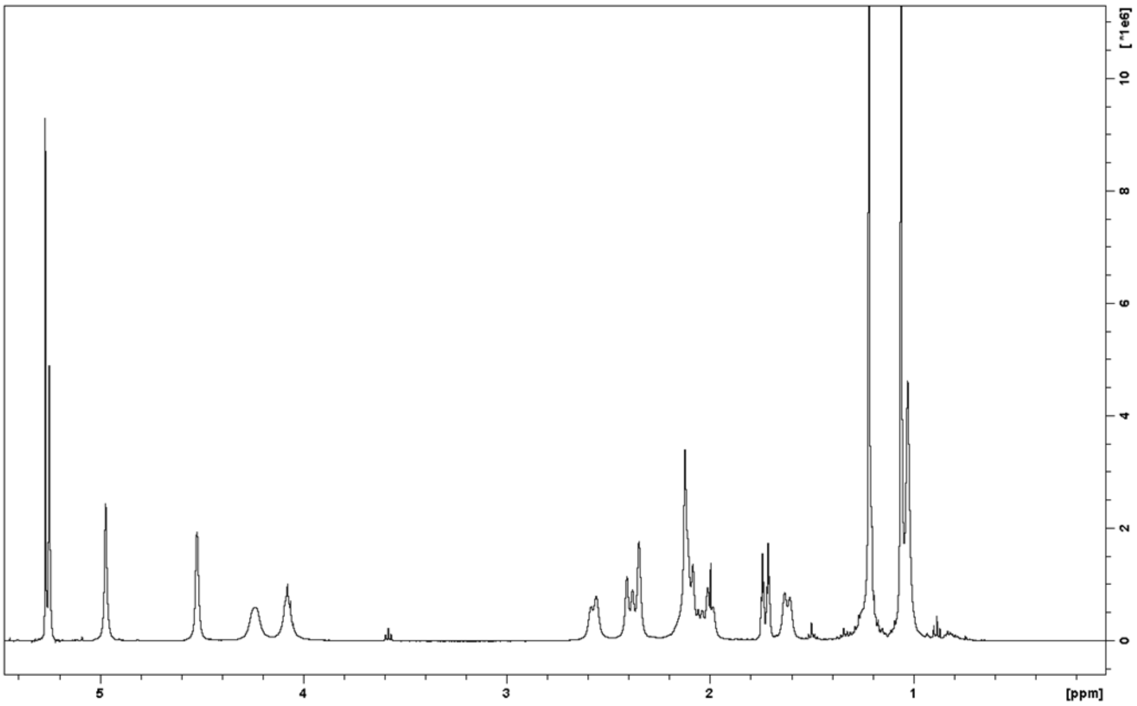

Figure S10. ^{13}C-NMR (125 MHz, $CDCl_3$) spectrum of compound **2**.

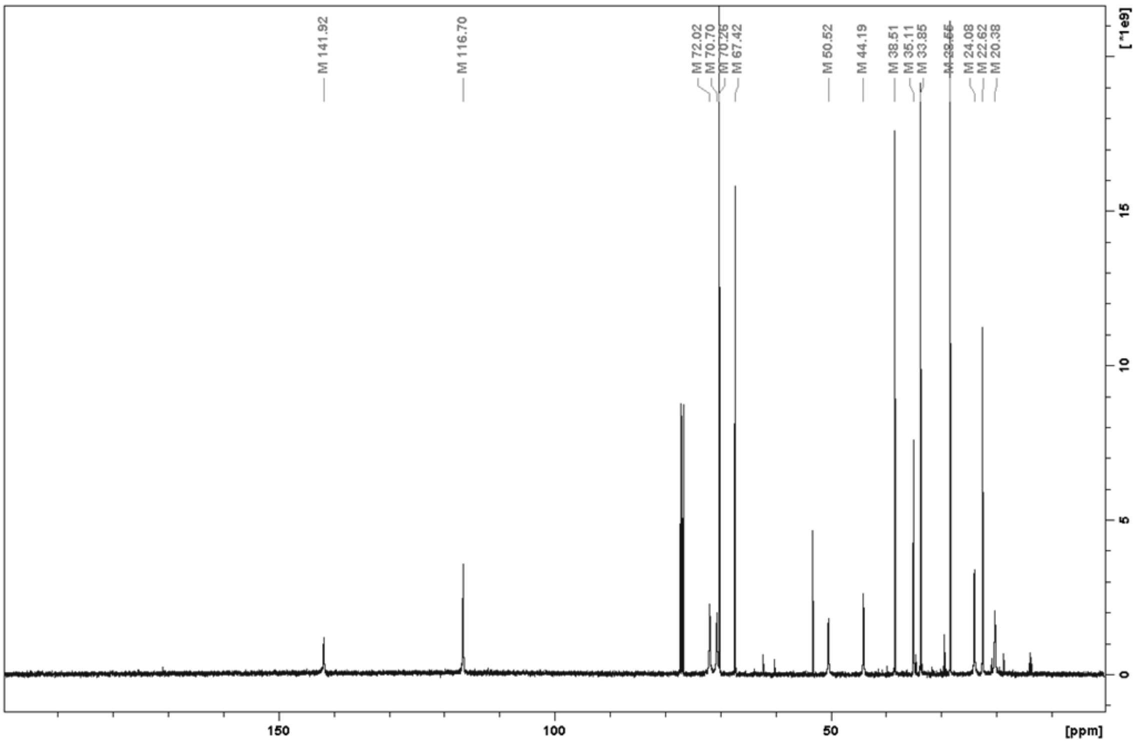

Figure S11. DEPT-135 NMR (125 MHz, $CDCl_3$) spectrum of compound **2**.

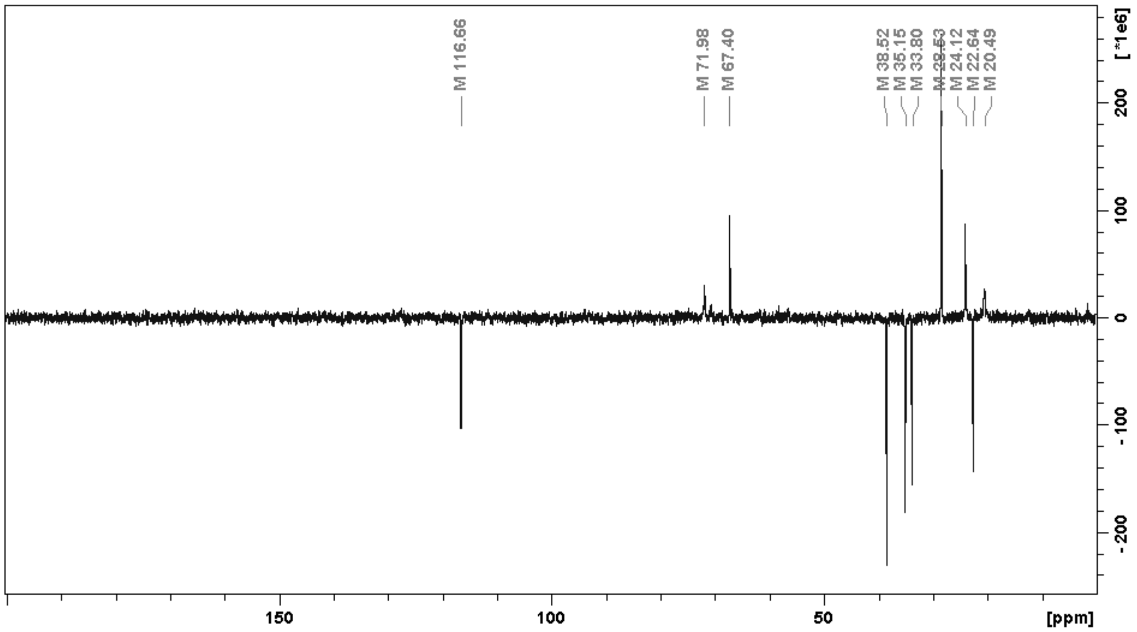

Figure S12. HSQC NMR (125 MHz, $CDCl_3$) spectrum of compound **2**.

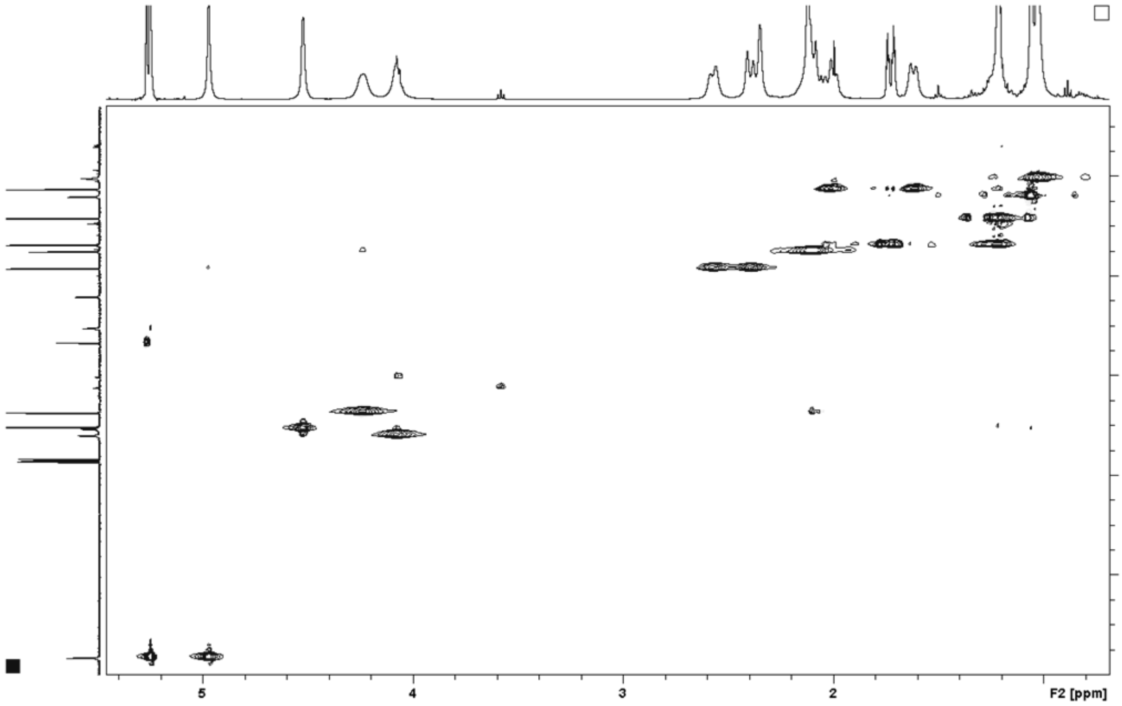

Figure S13. HMBC NMR (125 MHz, $CDCl_3$) spectrum of compound **2**.

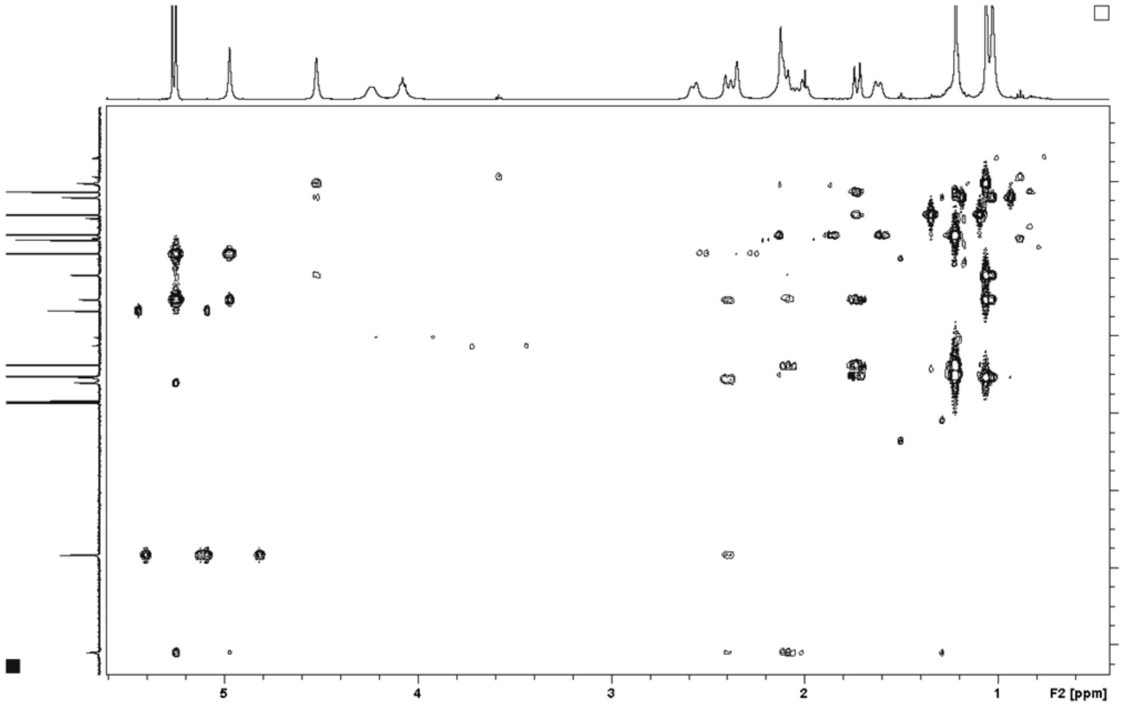

Figure S14. COSY NMR spectrum of compound **2**.

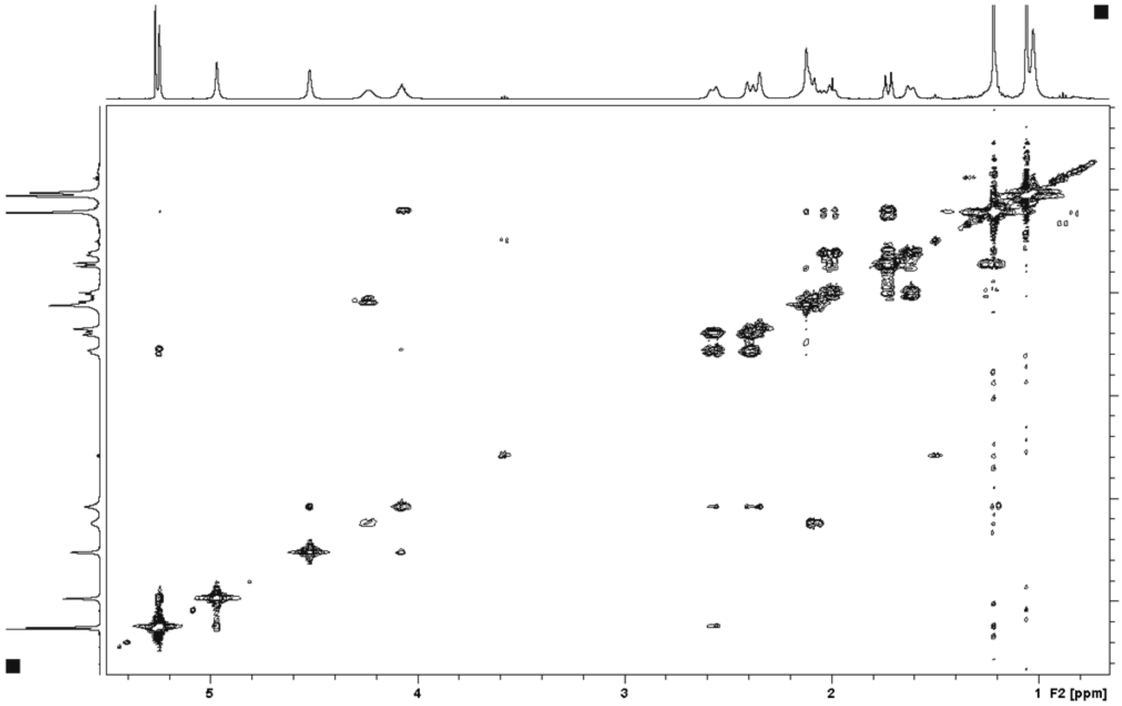

Figure S15. NOESY NMR spectrum of compound **2**.

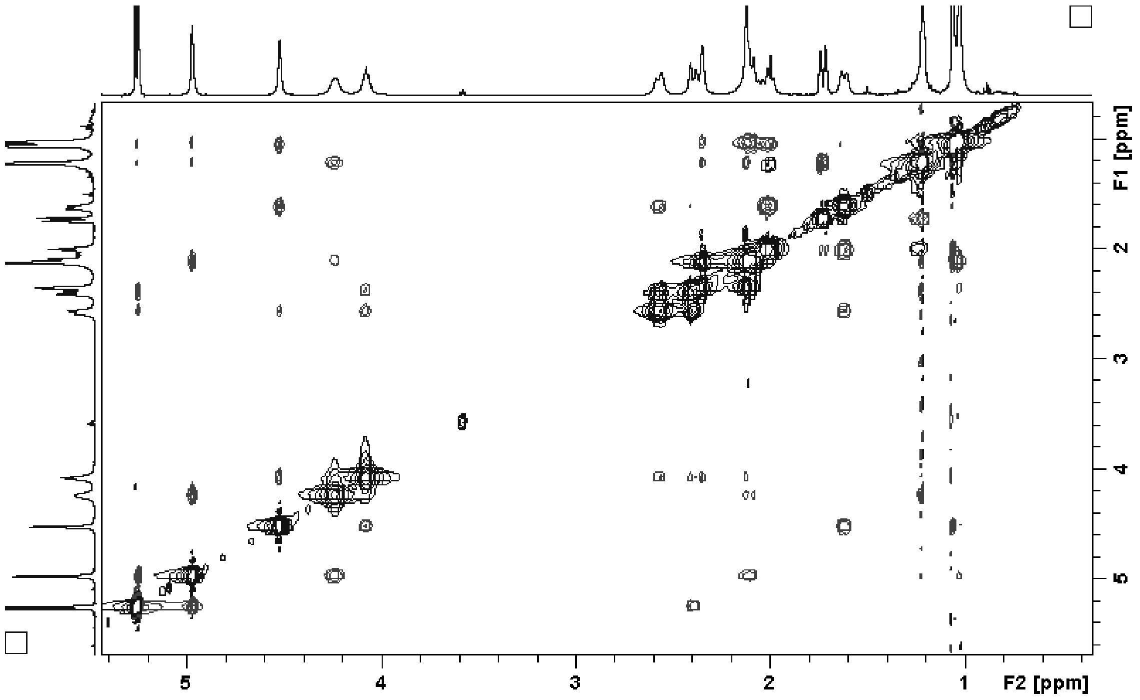

Figure S16. IR spectrum of compound **2**.

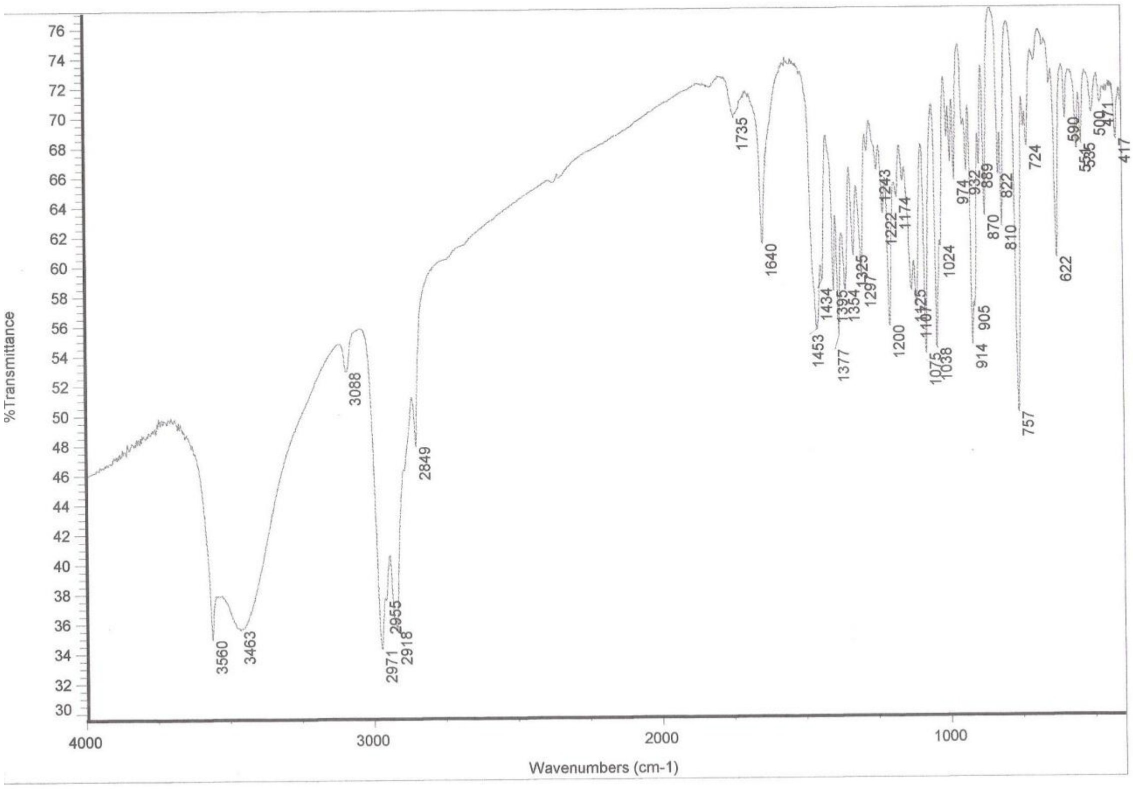

Figure S17. HR-ESI-MS (**A**) and EI-MS (**B**) spectra of compound **2**.

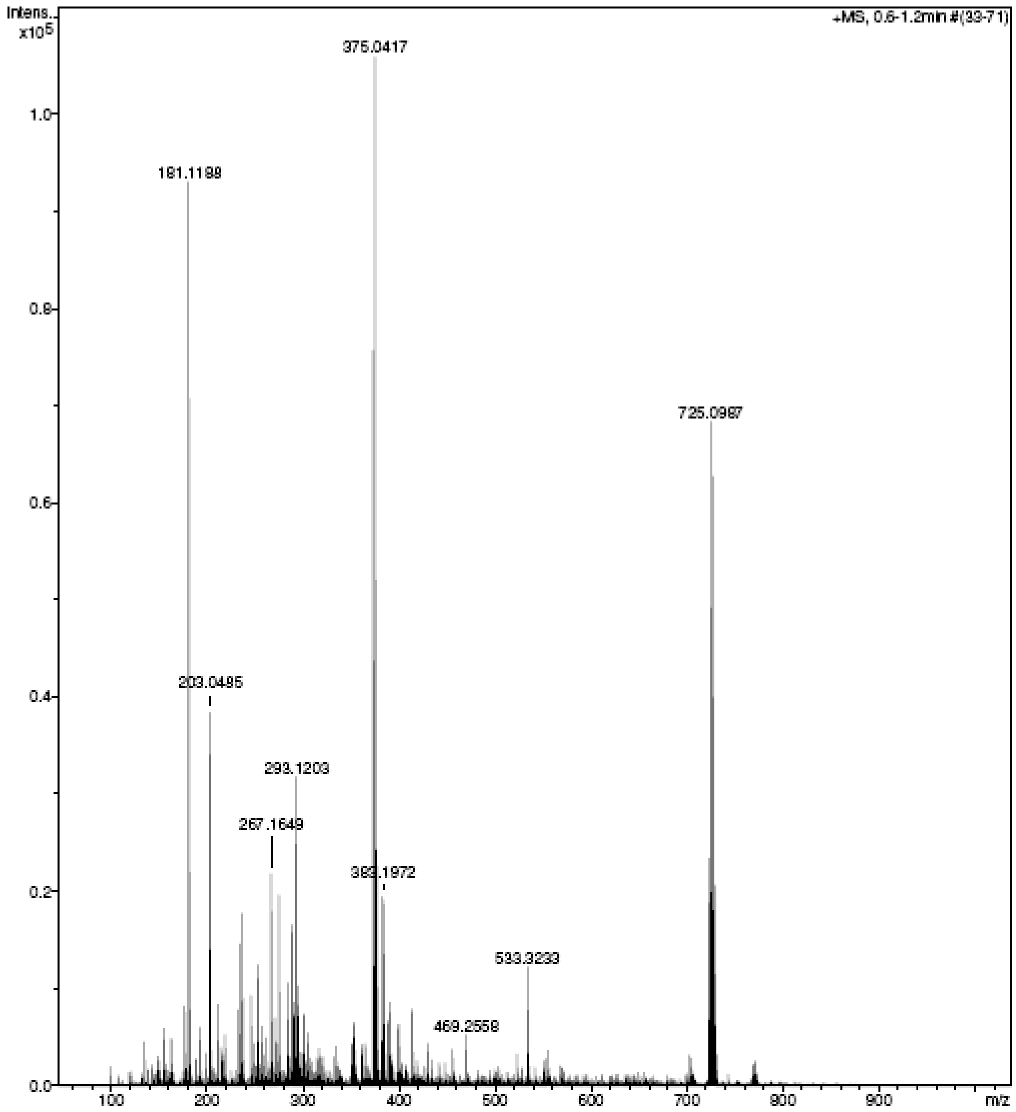

(**A**)

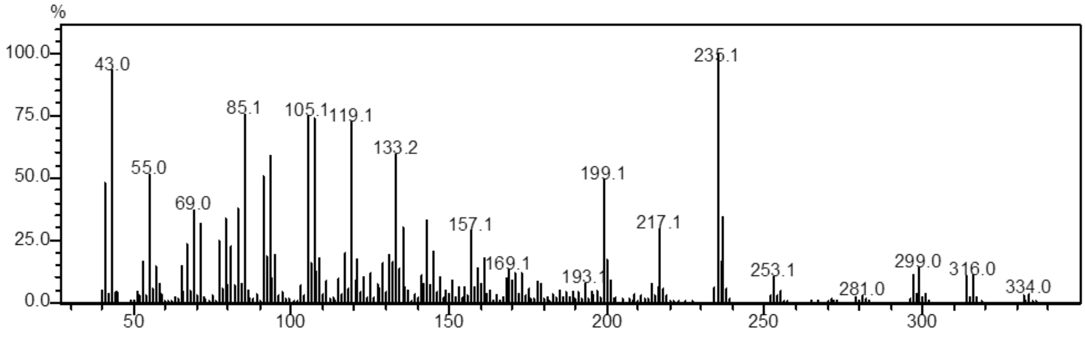

(**B**)

Figure S18. ^{1}H-NMR (500 MHz) spectrum of compound **3**.

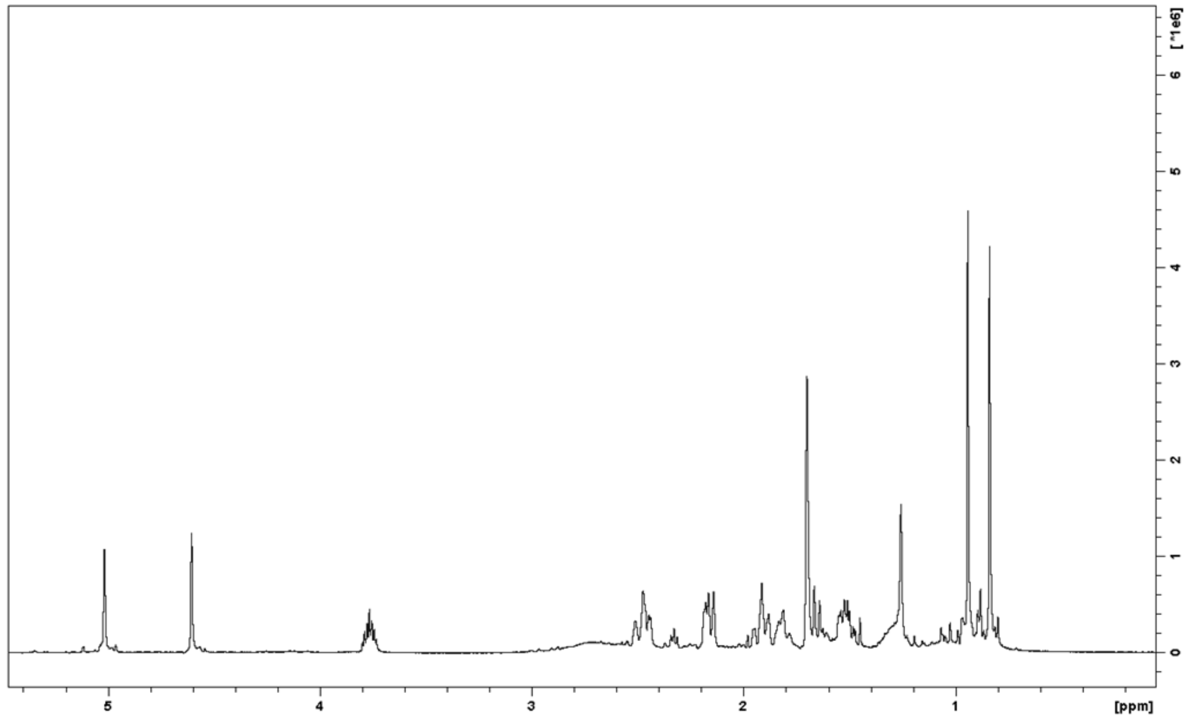

Figure S19. ^{13}C-NMR (125 MHz) spectrum of compound **3**.

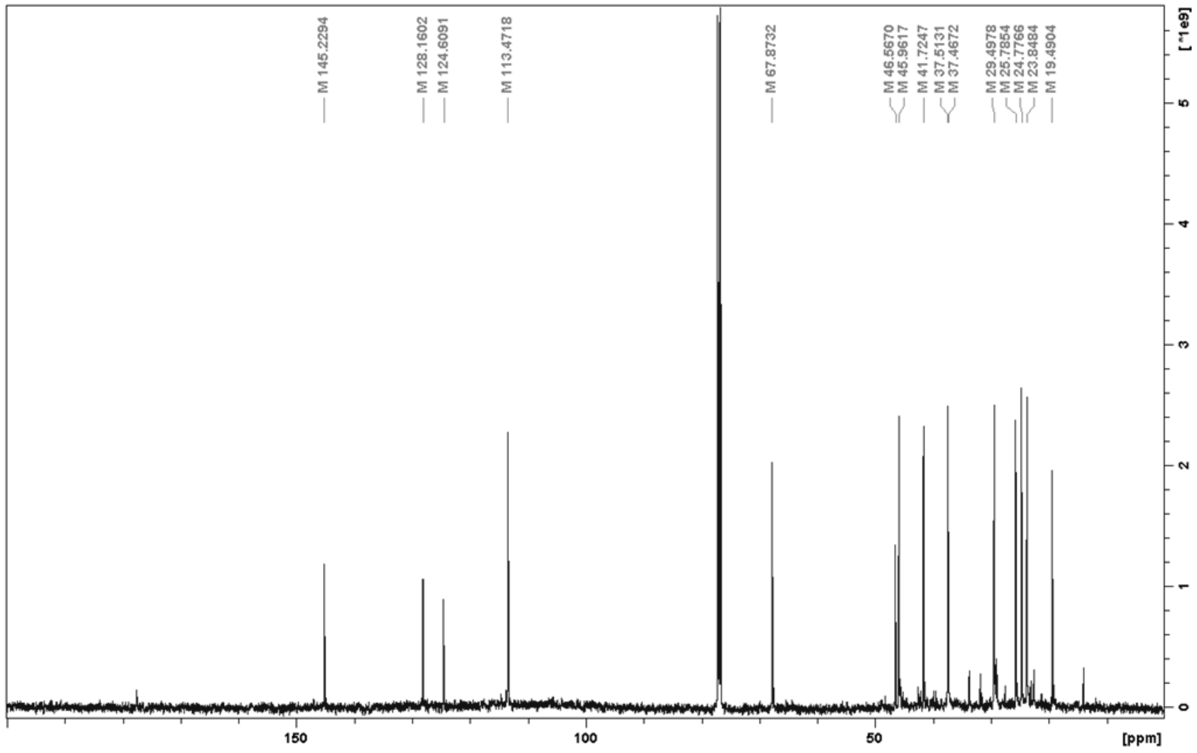

Figure S20. HSQC NMR (125 MHz) spectrum of compound **3**.

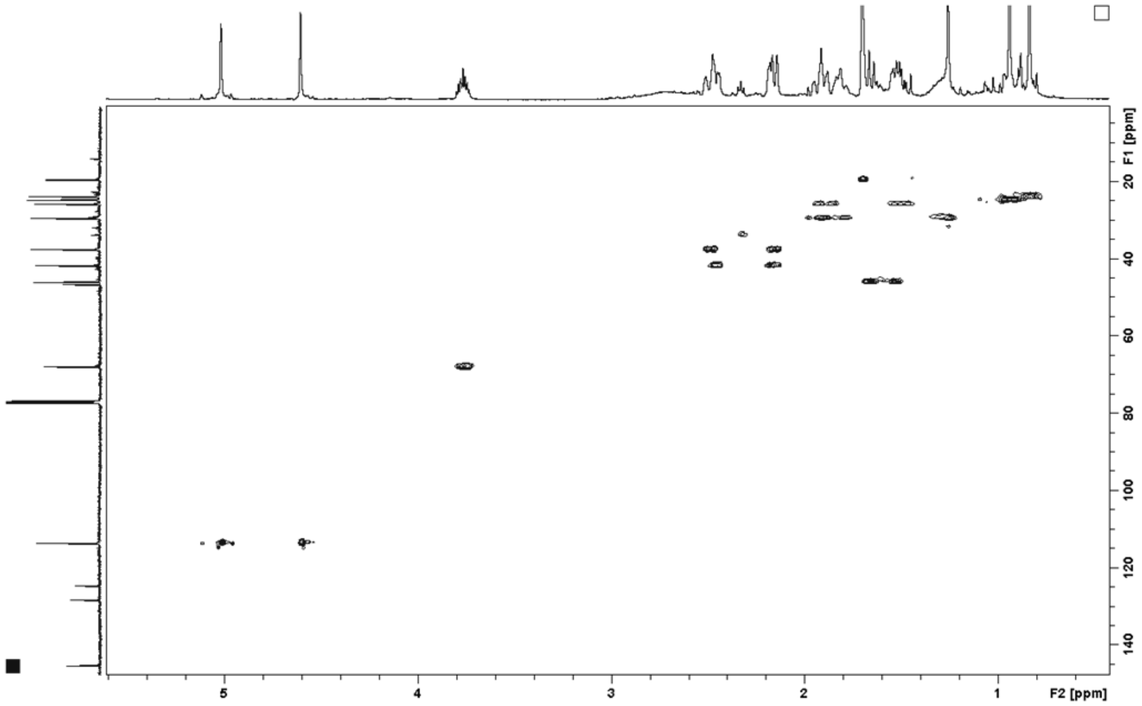

Figure S21. HMBC NMR (125 MHz) spectrum of compound **3**.

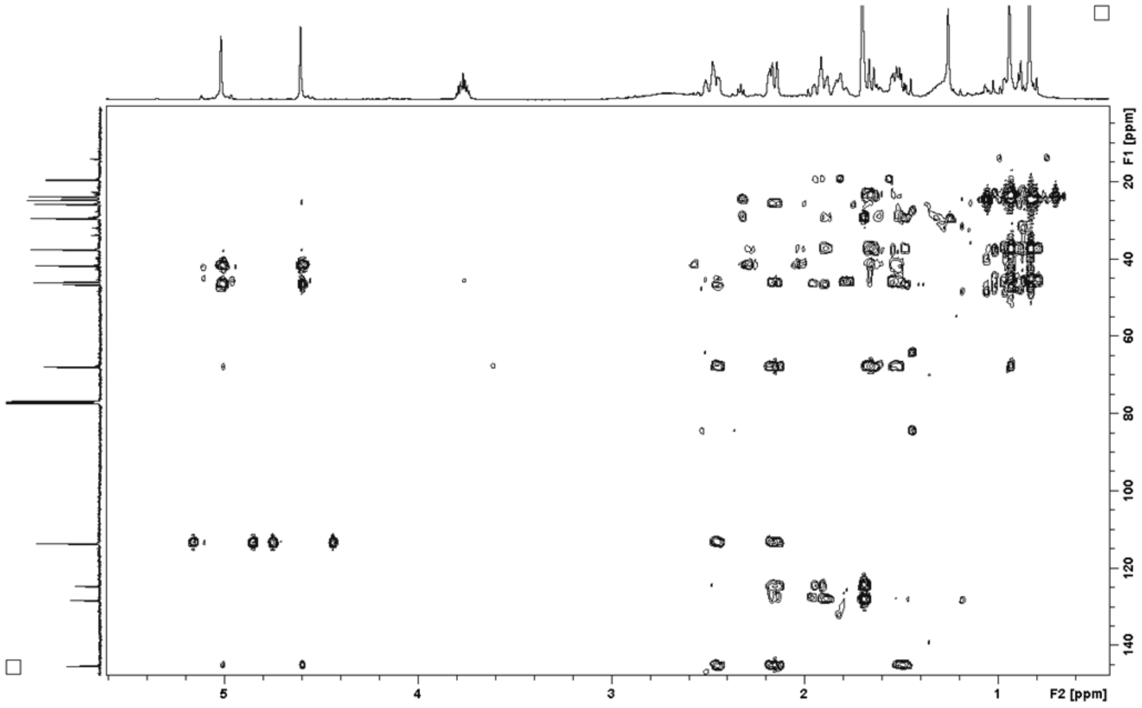

Figure S22. COSY NMR spectrum of compound **3**.

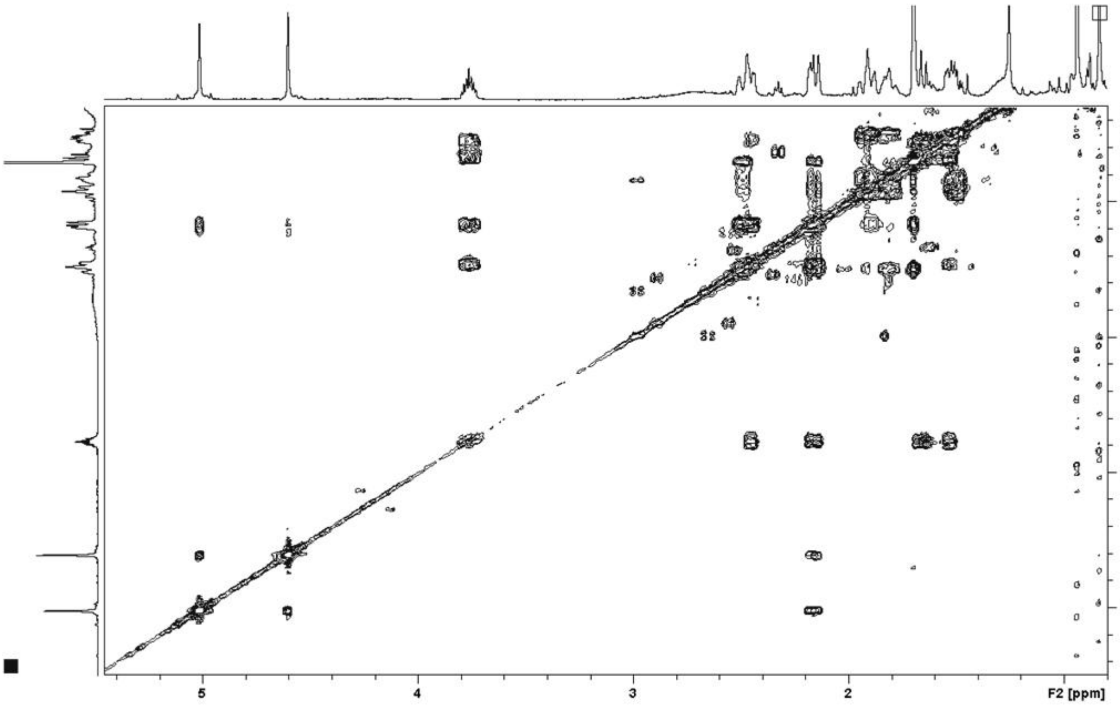

Figure S23. IR spectrum of compound **3**.

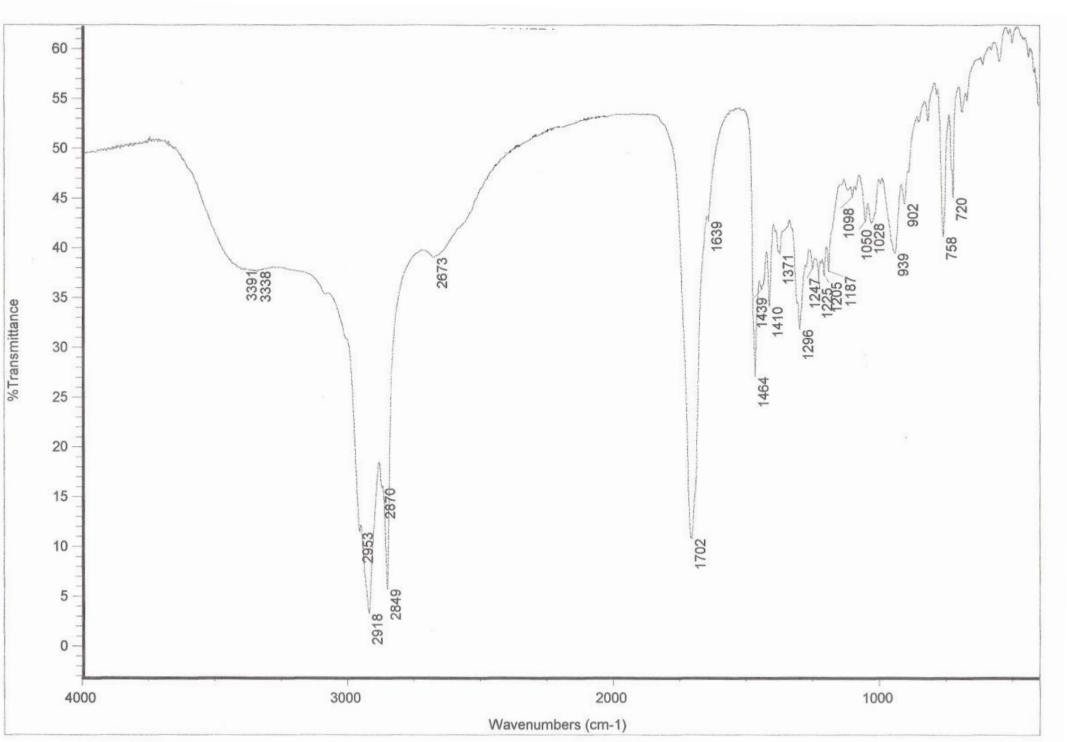

Figure S24. EI-MS spectrum of compound **3**.

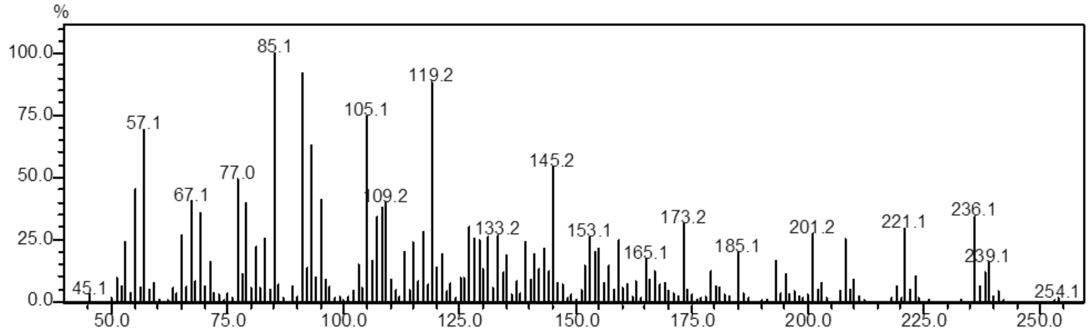

Table S1. ^{1}H- (300 MHz) and ^{13}C- (75 MHz) NMR data for compounds **4** and **5** ($CDCl_3$, δ in ppm).

**Position**	**4**		**5**	
	**δ$_C$**	**δH, mult. (*J* in Hz)**	**δ$_C$**	**δH, mult. (*J* in Hz)**
1	25.5	1.87 m<br>1.71 m	69.9	-
2	40.4	2.26 m<br>2.17 m	85.3	-
3	68.4	-	41.0	1.62 m
4	67.9	4.71 dd (12.4, 4.7)	47.3	1.61 m<br>1.34 m
5	37.2	2.27 m<br>1.96 dd (14.4,12.6)	49.7	-
6	50.4	-	41.8	1.46 m<br>1.46 m
7	145.7	-	29.0	1.51 m<br>1.29 m
8	33.5	2.32 m<br>2.16 m	57.7	1.50 m
9	35.8	2.27 m<br>2.05 m	42.5	1.38 m
10	63.5	4.44 dd (12.7, 4.5)	36.3	1.72 ddd (11.4, 5.7, 2.3)<br>1.15 dt (11.4, 6.9)
11	43.9	-	27.9	1.86 ddd (13.5, 6.9, 2.3)<br>1.42 m
12	23.5	1.14 s	22.3	1.10 s
13	17.4	0.96 s	12.5	0.87 d (6.1)
14	114.8	5.26 s<br>4.87 s	26.7	1.07 s
15	23.8	1.83 s	19.5	0.97 d (6.4)

Experimental Data of Known Compounds **4** and **5**.

Obtusane (**4**)*:* White gum; $[\alpha]_D^{25}$ +18.8 ° (*c* 0.05, $CHCl_3$); IR (KBr) ν_{max} 2926, 1716, 1456, 911, 870, 804, 738 cm^{-1}; ^{1}H-NMR and ^{13}C-NMR data, see Table 1S; EI-MS (rel. int.) *m/z* 402 (1), 400 (4), 398

(5), 396 (2), 385 (3), 383 (5), 381 (2), 319 (11), 318 (15), 316 (10), 283 (21), 281 (22), 239 (10), 237 (28), 202 (12), 201 (54), 109 (100), 107 (39).

(1*S**,2*S**,3*S**,5*S**,8*S**,9*S**)-2,3,5,9-Tetramethyltricyclo[6.3.0.0$^{1.5}$]undecan-2-ol (**5**): Colourless oil; $[\alpha]_D^{25}$ −11.3 ° (*c* 0.67, $CHCl_3$). IR (mineral oil) ν_{max} 3035, 2932, 2865, 1720, 1457, 1375, 1239, 1165, 1082, 1006, 900 cm^{-1}; ^{1}H-NMR and ^{13}C-NMR data, see Table 1S; EI-MS (rel. int.) *m/z* 222 (3), 207 (1), 189 (1), 135 (37), 86 (100), 85 (14), 81 (32), 79 (10).
